# Plant-derived nanovesicles: harnessing nature's power for tissue protection and repair

**DOI:** 10.1186/s12951-023-02193-7

**Published:** 2023-11-24

**Authors:** Xiaohang Chen, Xiaojie Xing, Shuoqi Lin, Liyu Huang, Lianghang He, Yuchun Zou, Xuyang Zhang, Bohua Su, Youguang Lu, Dali Zheng

**Affiliations:** 1 Fujian Key Laboratory of Oral Diseases, School and Hospital of Stomatology, Fujian Medical University, Fuzhou, China; 2 Department of Preventive Dentistry, School and Hospital of Stomatology, Fujian Medical University, Fuzhou, China; 3 Department of Human Anatomy and Histology, and Embryology, School of Basic Medical Sciences, Fujian Medical University, Fuzhou, China

## Abstract

Tissue damage and aging lead to dysfunction, disfigurement, and trauma, posing significant global challenges. Creating a regenerative microenvironment to resist external stimuli and induce stem cell differentiation is essential. Plant-derived nanovesicles (PDNVs) are naturally bioactive lipid bilayer nanovesicles that contain proteins, lipids, ribonucleic acid, and metabolites. They have shown potential in promoting cell growth, migration, and differentiation into various types of tissues. With immunomodulatory, microbiota regulatory, antioxidant, and anti-aging bioactivities, PDNVs are valuable in resisting external stimuli and facilitating tissue repair. The unique structure of PDNVs provides an optimal platform for drug encapsulation, and surface modifications enhance their stability and specificity. Moreover, by employing synergistic administration strategies, PDNVs can maximize their therapeutic potential. This review summarized the progress and prospects of PDNVs as regenerative tools, provided insights into their selection for repair activities based on existing studies, considered the key challenge for clinical application, and anticipated their continued prominent role in the field of biomedicine.

**Graphical Abstract:**

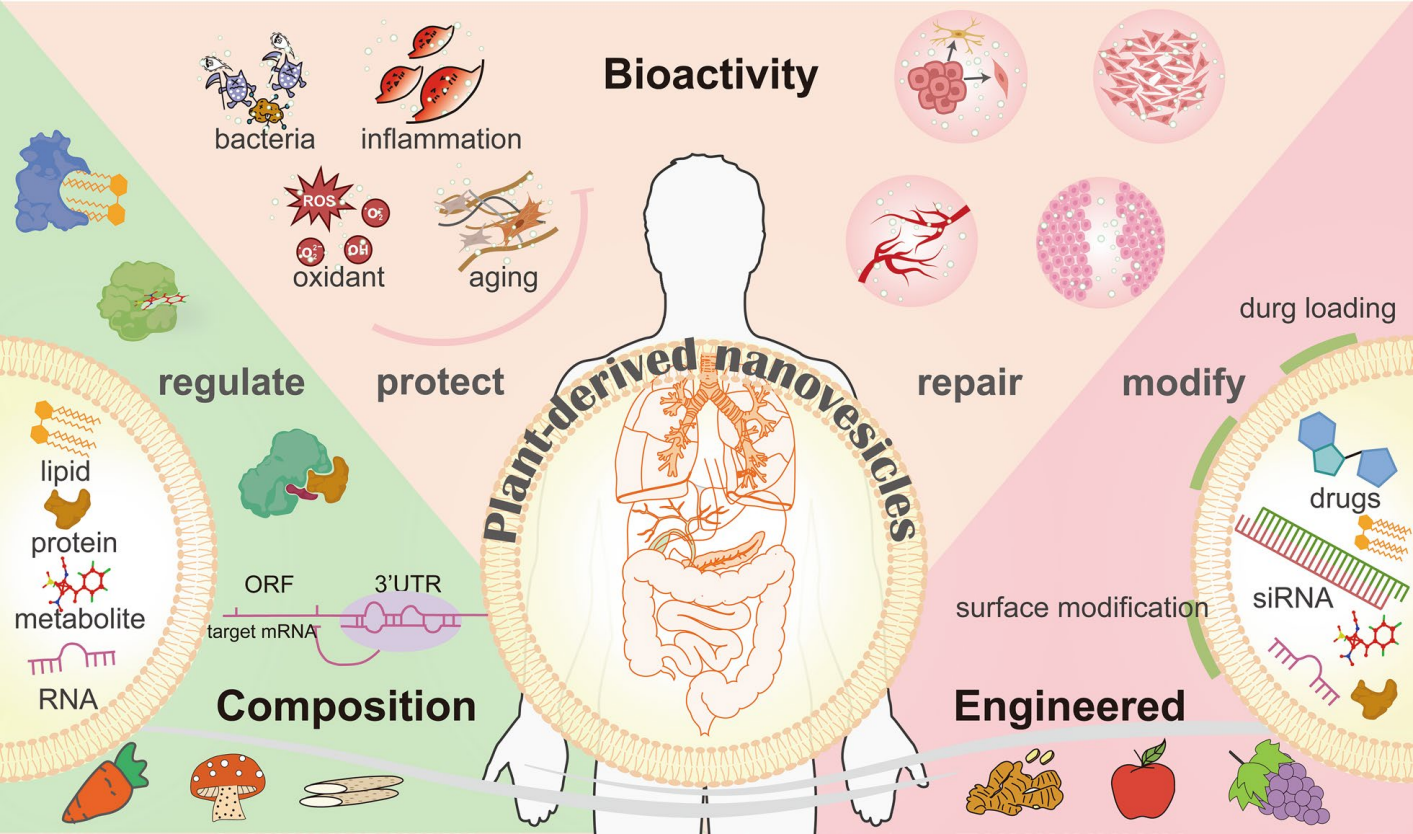

## Highlights

1. PDNVs, containing RNA, proteins, lipids, and metabolites, possess significant therapeutic bioactivity for tissue protection and repair.
2. Surface modification, drug loading, and optimal administration can enhance the therapeutic effects of PDNVs.
3. Exploring the relationship between the constituents and bioactivity of PDNVs holds potential for innovative therapeutic strategies in tissue damage repair.

## Introduction

Amidst the dazzling progress of modern civilization, society now faces challenges such as the relentless growth of the aging population and the spiraling incidence of diseases like obesity, and diabetes [1, 2]. These conditions lead to tissue aging, damage, and even loss, casting a shadow on the global economy and the well-being of humanity [3]. The lingering concern over tissue injury is primarily attributed to three main factors: (1) Lack of intervention: There are no effective measures to prevent various risk factors causing tissue damage such as the prevalence of obesity (with 603.7 million adults globally in 2015 [4]) and diabetes (with a 6.1% incidence rate in 2021 [5]), leading to chronic damage to multiple tissues; (2) Potential treatment risks: Although various conventional (such as organ transplantation) and novel treatment methods (such as tissue regeneration) are available, they present numerous therapeutic risks, including surgical infections, bleeding, immune rejection, and undirected differentiation of stem cells [6]; (3) Low economic benefits: Current methods for repairing tissue damage after injury are often burdened with adverse side effects and high costs [7, 8].

In the face of these persistent and troubling issues, there is an urgent need arises for an effective and economically viable treatment approach. Extracellular vesicles derived from various mammalian cell sources serve as communication tools between cells, carrying RNA, proteins, lipids, and other cellular components. They demonstrate tremendous potential in various fields of tissue repair and protection [9, 10]. A significant challenge they encounter is their low cost-effectiveness, as the isolation of extracellular vesicles from cell culture supernatant is typically time-consuming and laborious [11]. Conversely, extracellular vesicles sourced from plants, given their wide availability, may offer readily accessible, cost-effective, and low-risk solutions [12]. Plant-derived extracellular vesicles serve as essential intercellular communication tools involved in plant growth, development, and combating external pathogen stimuli. In this review, we differentiate ‘‘plant-derived nanovesicles (PDNVs)’’ from plant-derived extracellular vesicles, as existing isolation methods often co-isolate other vesicle-like structures, such as thylakoid [13].

PDNVs are widely sourced from the natural world, indicating higher economic benefits compared to animal-derived extracellular vesicles [14, 15]. Additionally, similar to mammalian extracellular vesicles, PDNVs isolated from plants consist of a lipid bilayer, containing proteins, lipids, and ribonucleic acid (RNA). However, due to their plant origin, PDNVs also contain various secondary metabolites not found in mammalian extracellular vesicles [16]. These diverse constituents collectively confer distinct natural bioactivity to PDNVs. Although the composition of PDNVs may not be identical to that of their plant sources due to the potential sorting mechanism of extracellular vesicles [17], extensive research on plants suggests that PDNVs may exhibit specific biological activities and serve as natural delivery tools for drug delivery. For example, cucumber-derived nanovesicles may contain cucurbitacin B [18], while nanovesicles from lemons and apples may contain various flavonoids [16]. This indicates that PDNVs naturally carry specific components found in plants and can exhibit corresponding natural bioactivity.

PDNVs are expected to become a promising strategy for tissue protection and repair due to their natural characteristics. (1) Natural bioactivity: PDNVs could be easily absorbed, promoting cell proliferation [19], inducing cell differentiation [20], and resisting external stimuli [21]. These properties provide a favorable microenvironment for tissue protection and repair. (2) Natural drug delivery tools: PDNVs naturally encapsulate a wide range of natural drugs, imparting a "protective shield" effect that enhances their bioavailability [22]. Furthermore, unlike conventional synthetic drug delivery systems, PDNVs function as drug carriers without requiring complex preparation processes. They serve as natural drug carriers, offering both surface and internal compartments for drug loading [22, 23]. (3) Natural targeting properties and modifiability: Different PDNVs with distinct compositions are selectively absorbed by specific cells [24], bacteria [25], and tissues [25]. Moreover, based on their vesicular structure, the surface of PDNVs provides numerous sites for further targeted modifications [26]. (4) Natural penetrability: PDNVs can cross the cell barrier, blood–brain barrier, and skin barrier to protect or repair the tissues [24, 27]. (5) Biocompatibility: PDNVs, derived from natural sources and even part of our daily diet, have low immunogenicity and exhibit good biocompatibility [28].

Based on the numerous advantages of PDNVs, their potential in tissue protection and repair is promising [19, 25]. Notably, there are several aspects that require exploration and summarization regarding PDNVs: (1) identifying the specific components in PDNVs that contribute to tissue protective and repair effects, (2) understanding how PDNVs regulate cellular behavior for tissue protection and repair, (3) exploring the potential applications of PDNVs in specific organs and tissues, (4) developing strategies to maximize the utilization of PDNVs' natural properties, including surface modification and drug loading, (5) selecting desired PDNVs from a wide range of plant species, and (6) presenting the challenges associated with the clinical application of PDNVs. Therefore, we have conducted a comprehensive review around these six questions, with the aim of further establishing PDNVs as valuable tools for tissue protection and repair.

## Active composition of PDNVs in tissue protection and repair

### miRNAs

MicroRNAs(miRNAs), a class of non-coding RNA species approximately 20–25 nucleotides in length, play a crucial role in various biological processes, including development, differentiation, proliferation, and apoptosis [29]. By binding with target messenger RNA (mRNA), miRNA can inhibit mRNA translation or promote mRNA degradation, thereby regulating gene expression [29]. Of particular interest is their presence in PDNVs, which are capable of intercellular communication for plants to resist external stimulation and maintain normal growth [30].

In the context of PDNVs, miRNAs have been demonstrated to effectively penetrate human cells and exert functional effects. A comprehensive study focusing on miRNAs derived from 11 different PDNVs has revealed that their target genes primarily participate in the regulation of inflammation-related pathways [31]. Notably, Peng and colleagues found that co-culturing bone marrow mesenchymal stem cells (BMSCs) with ginseng-derived nanovesicles resulted in the internalization of numerous miRNAs from the PDNVs into the BMSCs. These specific miRNAs were found to target genes associated with the regulation of neural differentiation. Furthermore, the researchers observed a remarkable enhancement of neural differentiation in BMSCs when exposed to ginseng-derived nanovesicles [20]. These cumulative findings strongly indicate that miRNAs present in PDNVs could serve as crucial active components involved in tissue protection and repair.

During inflammation and infection processes, TNF-α and LPS serve as the primary culprits causing tissue damage, while PDNVs have the potential to protect cells from their detrimental effects. Blueberries-derived nanovesicles containing miR-156a, miR-162, and miR-319d showed the potential as protector of human stabilized endothelial cell line (EA.hy926) for combating the external stimuli of tumor necrosis factor-α (TNF-α) [32]. Similarly, the miR-7972 in Rehmannia-derived nanovesicles could target G protein-coupled receptor 161 (GPR161), inhibiting the inflammatory factors secretion after lipopolysaccharide (LPS) stimulation and promoting the transformation of macrophages from pro-inflammatory M1 to anti-inflammatory M2. These miRNAs, enriched in PDNVs, exert their protective effects by targeting specific cellular pathways involved in inflammation and promoting cellular resilience.

The regulatory role of miRNA within PDNVs in maintaining the homeostasis of the microbiota is equally important for tissue protection and repair. miR-7972 derived from Rehmannia-derived nanovesicles could influence the balance of intestinal flora and inhibit the secretion amount of shiga toxin 2 -neutralizing factor (stx2), which plays a crucial role in the pathogenicity of enteric bacteria such as Escherichia coli (*E. coli*) [33]. Moreover, buckwheat-derived nanovesicles containing miR-6300 have shown the potential to promote the formation of *E.coli* biofilms, which can have implications in both pathogenicity and antimicrobial resistance [34]. Additionally, ginger-derived nanovesicles, enriched with miRNAs such as aly-miR-159a and gma-miR-166, have been found to weaken the virulence of *Porphyromonas gingivalis* (*P. gingivalis*) and reduce its adhesion ability to gingival epithelial cells [35]. These findings highlight the diverse and profound effects of PDNVs and their associated miRNAs in modulating inflammatory responses, cellular behavior, and microbial interactions. It is worth noting that there is still a need for further research on the effects of miRNA in PDNVs. Many studies have only predicted the tissue-protective and reparative potential of certain miRNAs, but direct evidence is lacking.

### Lipids

As fundamental biomolecules, lipids play diverse and essential roles in the functioning of living organisms [36]. Lipids in PDNVs, which resemble liposomes in structure, contribute significantly to the bioactivity and biocompatibility of these nanovesicles, thereby influencing their therapeutic effects [30]. The lipid content in PDNVs varies according to their plant source. For example, ginger-derived nanovesicles contain a significantly higher percentage of phosphatidic acid (PA) (47.2 ± 5.2%) compared to grape-derived nanovesicles (18.2 ± 1.9%) [37]. Different lipid compositions confer distinct functionalities and targeting capabilities to PDNVs.

On the one hand, lipids play crucial roles in the natural targeting and uptake of PDNVs by specific cells and bacteria. This targeting ability is exemplified by the diverse lipid compositions found in different types of PDNVs. For instance, grapefruit-derived nanovesicles utilize phosphatidylcholine (PC) to target the liver, while ginger-derived nanovesicles rich in PA tend to remain in the intestine [25]. Moreover, specific lipid components within PDNVs facilitate the selectively towards certain bacteria in a lipid-dependent manner [38]. For example, ginger and turmeric-derived nanovesicles, which are rich in PA, are preferentially taken up by *Lactobacillus reuteri*, whereas grapefruit and garlic-derived nanovesicles, rich in PC, are favored by *Ruminococcus sp.* [25]. However, the ratios of these lipids currently lack a systematic summary and explanatory principles.

On the other hand, the lipid components of PDNVs also exhibit tissue-protective and reparative effects. For instance, the PA present in ginger-derived nanovesicles can induce the phosphorylation and expression of forkhead box protein A2b (Foxa2) in intestinal epithelial cells, thereby influencing the secretion of exosomes to prevent insulin resistance [39]. Intriguingly, certain lipids have been found to possess anti-inflammatory properties. Digalactosydiacylglycerol (DGDG) within oats-derived nanovesicles would reduce inflammation by preventing the binding of β-glucan to Dectin-1 [24]. Additionally, the sulforaphane lipid in broccoli-derived nanovesicles may activate the mitogen-activated protein kinase (MAPK) signaling pathway in dendritic cells, enhancing their tolerance and preventing excessive inflammatory responses, thus offering protection against ulcerative colitis [40]. Moreover, garlic chives-derived nanovesicles have shown the ability to inhibit the activation of the NOD-like receptor family pyrin domain-containing protein 3 (NLRP3) inflammasome, with 1,2-diacyl-sn-glycerol-3-phosphate (DLPC) as the primary active substance [41]. These research findings suggest that the lipid components within PDNVs play a significant role in regulating cellular function and recognition. However, there are still many mechanisms that remain unclear.

### Metabolites

**Fig. 1:**
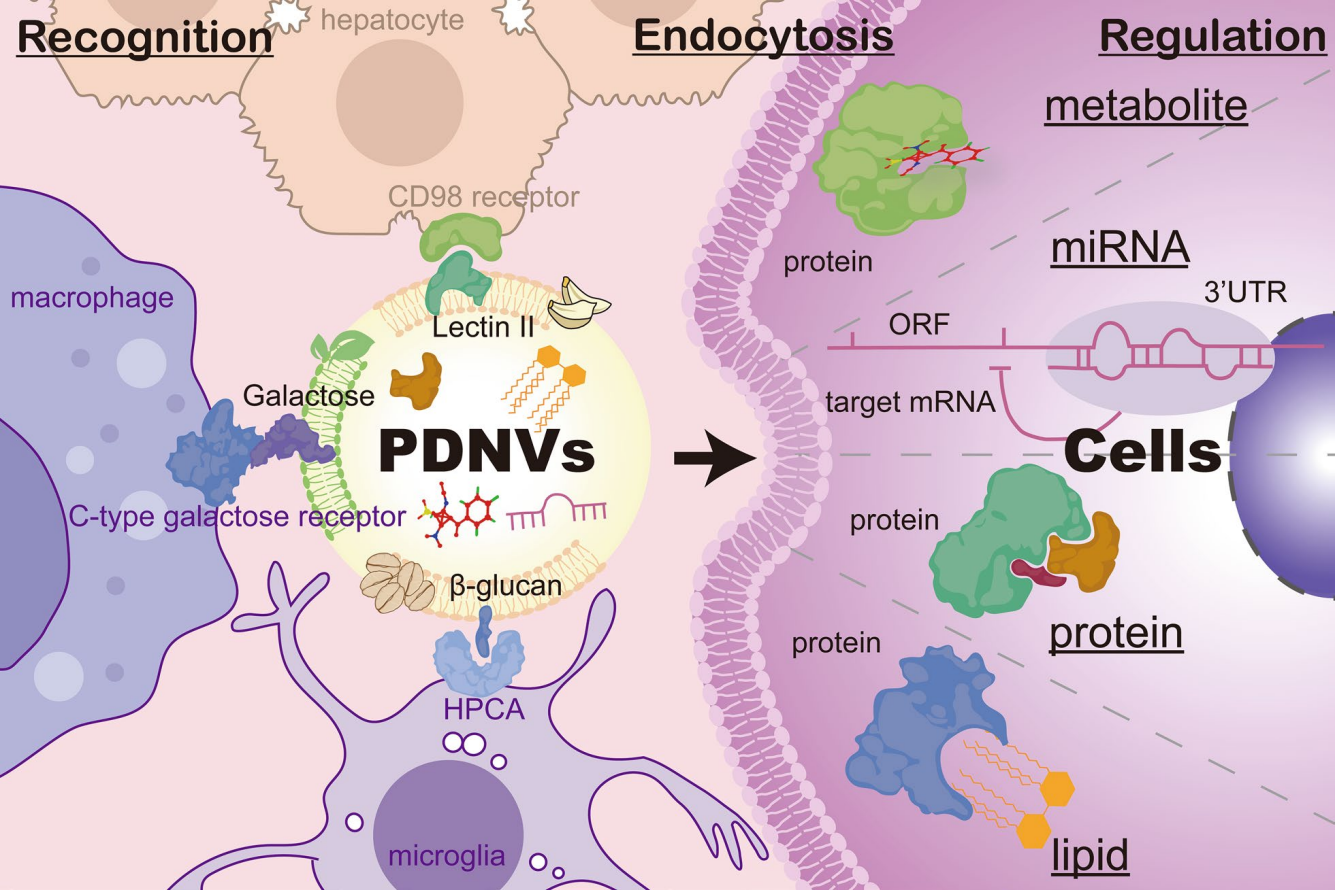
PDNVs composition and mechanism of action. The recognition and binding of β-glucan from oats-derived nanovesicles and HPCA from microglia [24]; galactose from tea-derived nanovesicles and the C-type galactose receptor from macrophages [43]; and Lectin II from garlic-derived nanovesicles and the CD98 receptor from liver cells [46]. (PDNVs, Plant-derived nanovesicles, ORF, open reading frame, HPCA, hippocampal calcium-binding protein, 3′UTR, 3′ untranslated region, CD98 receptor, cluster of differentiation 98 receptor.)

Plant metabolites constitute a significant portion of the components in PDNVs. Interestingly, these metabolites are sometimes present in higher concentrations within PDNVs compared to the original plant sources. For instance, ginger-derived nanovesicles contain elevated levels of lipophilic compounds such as 6-gingerol, 8-gingerol, and 10-gingerol [42]. The enrichment of these metabolites played a vital role in the bioactivity of PDNVs, enabling them to recognize and bind to specific cells. For example, oats-derived nanovesicles contain β-glucan, which binds to hippocampal calcium-binding protein (HPCA) on microglia, facilitating targeted endocytosis [24] (Fig. 1). Similarly, green tea-derived nanovesicles with galactose were recognized by the galactose receptor on macrophages [43], promoting their internalization (Fig. 1).

The interactions between specific active metabolites in PDNVs are also critical for their role in tissue protection and repair. For example, ginger-derived nanovesicles, abundant in shogaol, can regulate the expression of nuclear factor erythroid 2-related factor 2 (Nrf2) in primary hepatocytes, thereby modulating their antioxidant activity to protect against liver damage [44]. Meanwhile, lemon-derived nanovesicles enriched with pectic polysaccharides like rhamnogalacturonan enhance the stress survival of gut bacteria through RNase P-mediated degradation of specific tRNAs. The mechanism provides support and protection for the gut microbiota [45]. While many studies have demonstrated the tissue regeneration and repair properties of metabolites in PDNVs, the inheritance relationship between these metabolites and natural plants is not yet known. It is also unclear which PDNVs contain a higher abundance of the metabolites we desire.

### Proteins

Several studies have shed light on the significance of proteins in PDNVs concerning cell recognition and functionality, contributing to a deeper understanding of their mechanisms. For example, Song et al. conducted research on garlic-derived nanovesicles (GaNVs) and demonstrated the elimination of surface proteins using trypsin resulted in a substantial decrease in cellular uptake efficiency, highlighting their crucial role in endocytosis [46]. Additionally, it was observed that certain surface proteins of GaNVs colocalized with CD98 during the endocytosis process (Fig. 1). Furthermore, the blocking of the mannose-specific binding protein II lectin on GaNVs and the CD98 receptor hindered liver cell recognition [46]. Moreover, Sriwastva et al., revealed that a member of the heat shock protein family A 8 (HSPA8) and HSP70, derived from mulberry bark-derived nanovesicles, exhibited the ability to bind and activate the aryl hydrocarbon receptor (AhR) pathway, thereby preventing dextran sulfate sodium (DSS)-induced colitis. Remarkably, mulberry bark-derived HSPA8 displayed higher affinity for AhR compared to human-derived HSPA8 [47]. These findings highlight the necessity and tremendous prospects of proteins in PDNVs.

In conclusion, PDNVs harbor diverse active components with varying degrees of enrichment (Table 1). Due to variations in cell gene expression, the effects of PDNVs can differ across different cell types. The recognition mechanisms between cells and vesicles exhibit notable distinctions that warrant further investigation (Fig. 1). Unraveling these mechanisms holds immense potential in providing invaluable insights into precisely targeted delivery systems of natural origin. Moreover, it is important to note that PDNVs likely encompass a rich diversity of RNA species. While current research primarily focuses on exploring miRNA, other RNA molecules such as long non-coding RNA (lncRNA) and circular RNA (circRNA) remain largely unexplored. Intriguingly, some scholars have identified mitochondrial deoxyribonucleic acid (mtDNA) as a pivotal player within PDNVs, exerting a profound influence on reshaping the tumor-related immune microenvironment. These findings underscore the untapped potential of discovering additional active constituents within PDNVs [48].

**Table 1:** The key active ingredients and mechanisms of PDNVs in tissue protection and repair

Plant	Key active ingredients	Mechanisms	Activity	Refs.
Edible plant (ginger, grapefruit and others)	miR-530b	miR-530b targeted ribosomal slippage site in ORF1ab gene	Anti-virus	[96]
Peeled Hawaiian ginger roots	miR7267-3p; miR167a-5p	miR7267-3p mediated targeting of the LGG monooxygenase ycnE yields increased indole-3-carboxaldehyde (I3A). I3A activates AhR to induce the production of IL-22; miR167a-5p molecule targeted and reduced the expression of the LGG SpaC gene	Regulate microbial; anti-inflammatory	[25]
Ginger	Aly-miR396a-5p; rlcv-miR-rL1-28-3p	aly-miR396a-5p and rlcv-miR-rL1-28-3p mediated inhibition of expression of Nsp12 and spike genes	Anti-inflammatory; anti-virus	[89]
Ginger	Shogaol	Shogaol played a role in the induction of Nrf2 in a TLR4/TRIF-dependent manner	Anti-oxidant	[44]
Ginger	phosphatidic acid; miRNA	*P. gingivalis* were significantly reduced following interaction with including PA and miRs	Anti-bacterial	[35]
Ginger	Phosphatidic acid	Phosphatidic acid prevented phosphorylation of Foxa2 by inhibiting Akt-1 activation	Anti-inflammatory	[39]
Ginger Rhizomes	Lipid	It inhibited the NLRP3 inflammasome activity	Anti-inflammatory	[71]
Garlic	Phosphatidic acid (36:4)	Phosphatidic acid (36:4)/BASP1 led to inhibiting c-GAS/STING mediated expression of inflammatory cytokines via inhibition of the c-Myc activation in brain microglial cells	Anti-inflammatory	[72]
Garlic chive	phospholipid 1,2-dilinoleoyl-sn-glycero-3-phosphocholine	Phospholipid 1,2-dilinoleoyl-sn-glycero-3-phosphocholine in GC-VLNs has been identified to inhibit NLRP3 inflammasome activation	Anti-inflammatory	[41]
Mulberry bark	HSPA8	HSPA8 bound to and subsequently activated AhR and is required for the protective effect by inducing an array of anti‐microbial peptides	Anti-inflammatory	[47]
Oat	β-glucan	HPCA/β-glucan interaction facilitated exosome uptake by microglial cells. This action was recruited by Rab11A to the complex that a critical step for bringing Golgi dectin-1 to the recycling endosome and exosome pathways	Immunomodulation	[24]
Broccoli	Sulforaphane	Sulforaphane induces tolerogenic DCs via AMPK signaling	Immunomodulation	[40]
Lemon	galacturonic acid-enriched pectin-type polysaccharide	Galacturonic acid-enriched pectin-type polysaccharide downregulated tRNA_ser_^UCC^ and tRNA_ser_^UCG^ level to limiting production of Msp1 and Msp3 in the LGG	Increase bile resistance	[45]
Momordica charantia	miR5266	miR5266 could specifically inhibit MMP-9 by binding to its coding sequences within the 3′-UTR region	Protect the BBB integrity; preserve tight junction proteins	[87]
Rehmanniae Radix	miR-7972	miR-7972 downregulated the expression of GPR161, activating the Hedgehog pathway, and inhibited the biofilm form of Escherichia coli via targeting virulence gene sxt2	Anti-inflammatory; Anti-bacterial; Anti-oxidant	[33]
Apple	miRNA	miRNA reduced OATP2B1 expression in Caco-2 cells	–	[123, 124]
Ginseng	mtr-miR159a	miRNA might regulate the PI3K signaling and transcripts of BMSCs	Promote the neural differentiation	[20]
Strawberry	Vitamin C	Strawberry-derived nanovesicles prevented oxidative stress on human cells, possibly through the activity of vitamin C	Anti-oxidant	[81]
Catharanthus roseus	Probably lipids and proteins	They promoted macrophages to secrete a variety of cytokines and activate the inflammatory response via TNF-α/NF-κB/PU.1 axis	Immunomodulation	[117]

*ORF1b* open reading frame 1b, *LGG* *Lactobacillus* *rhamnosus* GG, *I3A* indole-3-carboxaldehyde, *AhR* the aryl hydrocarbon receptor, *IL-22* interleukin-22, *Nsp12* the non-structural protein 12 of coronaviruses, *Nrf2* nuclear factor erythroid 2-related factor 2, *TLR4* Toll-like receptors 4, *TRIF* TIR-domain-containing adaptor inducing interferon-β, *PA* phosphatidic acid, *Foxa2* forkhead box protein A2, *AKT1* AKT Serine/Threonine Kinase 1, *NLRP3* NOD-like receptor family pyrin domain-containing protein 3, *BASP1* brain acid soluble protein 1, *c-GAS* cyclic GMP-AMP synthase, *STING* stimulator of interferon genes, *GC-VLNs* garlic chive-derived vesicle-like nanoparticles, *HSPA8* heat shock protein family A (Hsp70) member 8, *HPCA* hippocampal calcium-binding protein; *Rab11A* ras-related protein rab-11A, *DC* dentritic cell, *MSP1/3* methylation-specific restriction endonuclease 1/3, *MMP-9* matrix metalloproteinase-9, 3′UTR, 3′ Untranslated Region; *OATP2B1* organic anion transporting polypeptides 2b1; *PI3K* the phosphatidylinositol3-kinase; *BMSC* Bone marrow mesenchymal stem cell; *TNF-α* Tumor Necrosis Factor-α, *NF-κB* nuclear factor kappa-B, *PU.1* spleen focus forming virus (SFFV)proviralintegration oncogene

## Bioactivity of PDNVs in tissue protection and repair

When tissues experience external stimuli such as trauma or infection, the body initiates a series of responses to maintain structure integrity and biological functions. This intricate process involves immune responses, defense against bacterial invasion, cell activation, proliferation, and differentiation [49]. Creating a favorable microenvironment for tissue protection and repair is vital, encompassing aspects such as ensuring blood supply, mitigating excessive inflammatory responses, preventing infections, and minimizing oxidative stress reactions [49].

**Fig. 2:**
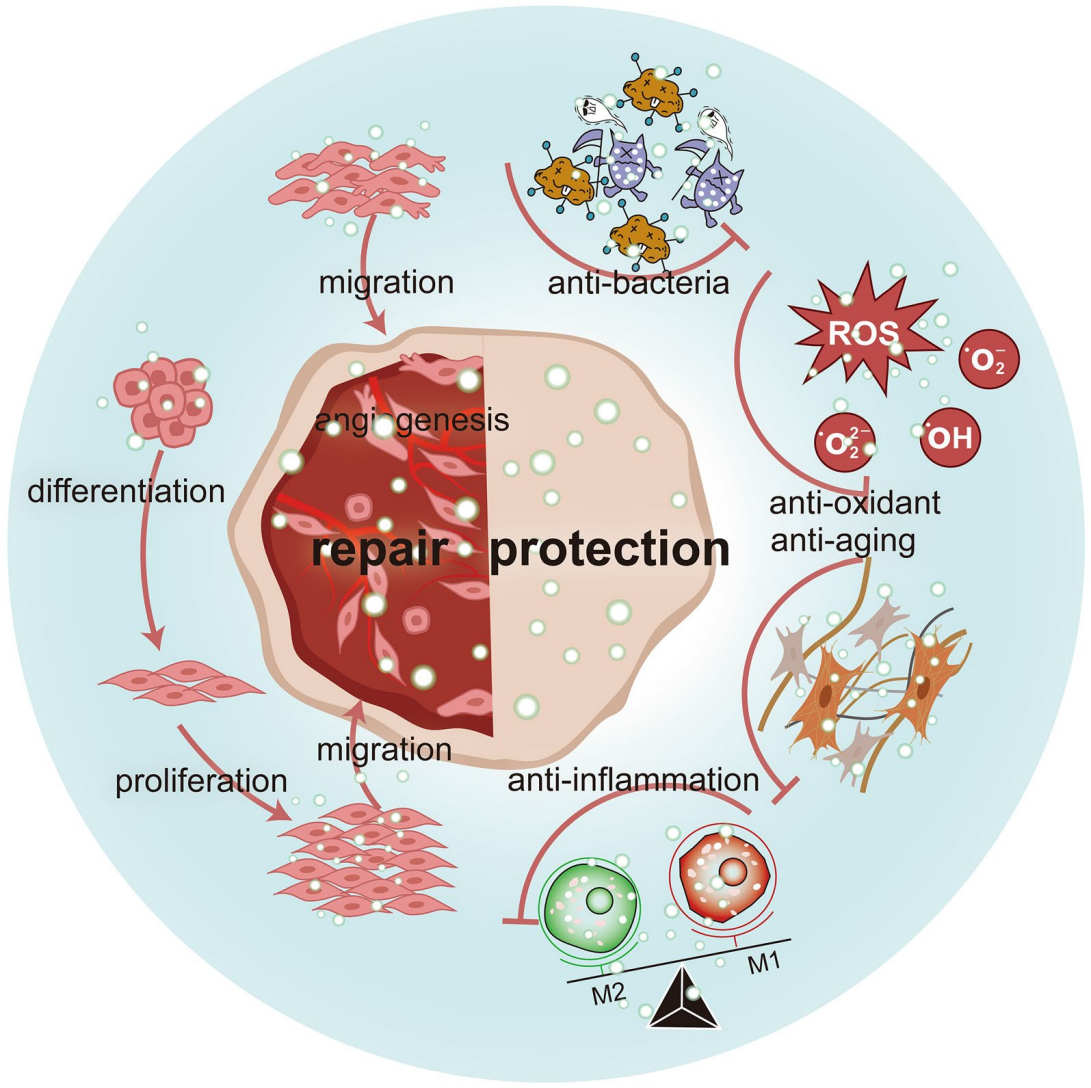
The bioactivity of PDNVs in tissue protection and repair. (*PDNVs* plant-derived nanovesicles, *ROS* reactive oxygen species.)

In this context, PDNVs exhibit intriguing therapeutic potential for tissue damage by offering a multifaceted approach. They have the ability to combat pathogens, promote cell proliferation [50], facilitate cell migration [51], induce cellular differentiation [19], stimulate angiogenesis [52], modulate immune responses [53], maintain microbial balance [35], and scavenge free radicals [54] (Fig. 2). The diverse range of these comprehensive properties in PDNVs makes them a promising tool in the field of tissue protection and repair.

### Enhancing cell proliferation

Cell proliferation plays a pivotal role in tissue healing. For instance, Aloe vera-derived nanovesicles have been shown to triple the proliferation potential of human dermal fibroblast cells [52]. In another study, apple-derived nanovesicles would significantly boost the proliferation of MC3T3-E1 osteoblasts [50]. Furthermore, bitter melon-derived nanovesicles demonstrated a dose-dependent promotion of cell proliferation, offering tissue protection during radiation exposure [55]. Studies have demonstrated that PDNVs possess the ability to enhance this process by activating specific cell signaling pathways. Similarly, ginger-derived nanovesicles have been found to stimulate the wingless/integrated-β-catenin (Wnt/β-catenin) signaling pathway, thereby augmenting the population of intestinal epithelial stem cells [37]. However, the mechanisms underlying the pro-proliferative effects of many PDNVs remain unknown.

It is important to note that the impact of PDNVs on cell proliferation is influenced by the source of nanovesicles [56]. Cabbage-derived nanovesicles were found to promote the proliferation of human adult epidermal keratinocyte cells (HaCaT) and mouse macrophage cell lines (RAW 264.7), but had no effect on HDFs. While red cabbage-derived nanovesicles exhibited a robust effect on proliferation regardless of the cell type [57]. Interestingly, certain PDNVs have demonstrated not only the ability to promote the proliferation of normal tissue cells but also to induce cell death in tumor cells [58]. For instance, garlic-derived nanovesicles showed significant cytotoxicity on A498 renal cell carcinoma cells and A549 lung carcinoma cells, while promoting the proliferation of normal fibroblasts at lower concentration [59, 60]. These findings not only highlight the biocompatibility of PDNVs but also suggest the existence of a potential natural target recognition switch, which warrants further investigation.

### Promoting cell migration

Cellular migration towards the site of injury plays a pivotal role in the process of tissue repair, representing a crucial step in the restoration of damaged tissues. On this context, the potential of PDNVs to positively influence cell migration have been observed through scratch assays, particularly utilizing aloe vera-derived nanovesicles [61, 62]. Notably, significant advancements in the healing rates of human dermal fibroblast cells have been achieved through the administration of high doses of 10^9^ aloe vera-derived nanovesicles, elevating the rates from 34 ± 4% and 48 ± 5% to 70 ± 8% and 100% respectively [52, 62]. Collagen I, as the main protein in the extracellular matrix, can provide support for cell migration. wheat-derived nanovesicles have demonstrated the ability to stimulate fibroblast migration and the secretion of type I collagen, with a notable effect observed at a concentration of 200 μg/mL [51]. Nevertheless, further comprehensive investigations are imperative to explore the in vivo effects of these nanovesicles and to elucidate the underlying mechanisms that contribute to their promotion of cell migration.

### Inducing cell differentiation

Cell differentiation is a complex process involving the morphological and functional transformation of cells, leading to the development of various cell types [63]. While this process is primarily associated with embryonic development, it also plays a crucial role in tissue repair and regeneration. Inducing directed cell differentiation is essential for organ injury repair.

#### Osteogenic differentiation

Certain active components derived from plants, such as diosgenin and dioscin from yam, and icariin from epimedium, have been shown to induce stem cell differentiation into osteoblasts, and PDNVs exhibit similar effects. Yam-derived nanovesicles have been found to stimulate osteogenic cell differentiation and mineralization by activating the bone morphogenetic protein-2(BMP-2)/phosphorylated P38 (p-P38)-dependent runt-related transcription factor 2 (Runx2) pathway [19]. Similarly, apple-derived nanovesicles modulate the BMP-2/mothers against decapentaplegic homolog 1 (Smad-1) pathway, enhancing the expression of osteogenic genes and proteins in MC3T3-E1 osteoblasts [50].

#### Chondrogenic differentiation

PDNVs have also demonstrated the ability to accelerate the chondrogenic differentiation process. For instance, tomato-derived nanovesicles have shown superior potential compared to the chondrogenic differentiation effects of lemon-derived nanovesicles in promoting stem cell chondrogenic differentiation. Tomato-derived nanovesicles significantly upregulate the expression of chondrogenic cell markers and critical proteins involved in the chondrogenic extracellular matrix of human adipose-derived mesenchymal stem cells [56].

#### Neural differentiation

Loss of sensation after injury healing can be disheartening. However, ginseng-derived nanovesicles have been found to stimulate neural differentiation in BMSCs by transferring miRNA that target genes enriched in neural-related signaling pathways. Treatment with ginseng-derived nanovesicles results in BMSCs displaying distinct electrophysiological characteristics and neuron-like extensions, possibly through the regulation of the phosphatidylinositol3-kinase (PI3K) signaling transduction and transcription [20]. These findings suggest that PDNVs has the potential to promote tissue regeneration through induced cell differentiation. However, further research is needed to fully elucidate the underlying mechanisms of action. It is important to emphasize that the undirected differentiation of stem cells can introduce uncertainty into tissue regeneration. Although PDNVs have exhibited promising potential in regulating cell differentiation, the lack of direct animal experimental evidence and safety assessment pertaining to their role in cell differentiation should be duly noted.

### Pro-angiogenic effect

The formation of new blood vessels, known as angiogenesis, is crucial for providing nutrients and removing waste during the possess of tissue healing [64]. Experimental studies have provided evidence for the pro-angiogenic effects of PDNVs. For instance, wheat-derived and beet juice-derived nanovesicles have been shown to support the formation of vascular-like networks by endothelial cells [65]. These nanovesicles demonstrated statistically significant improvements in both the length and number of branches in the network. Additionally, studies have revealed that the effect of aster yomena callus-derived nanovesicles on tube formation was concentration-related [66]. At lower concentrations of 0.1 and 1 × 10^9^/mL, the number of tubes increased initially and then decreased, while at a higher dose of 5 × 10^9^/mL, the number of tubes reduced [52]. Moreover, Peng's team has validated that ginseng-derived nanovesicles significantly enhance blood vessel formation during the rat wound healing process [20]. These findings highlight the ability of PDNVs to promote angiogenesis, which plays a critical role in reestablishing proper blood supply to the injured tissues. However, further investigations are necessary to fully understand the underlying mechanisms and optimize their therapeutic applications.

### Immunoregulation

While the immune system plays a crucial role in eliminating foreign pathogens and damaged cells, excessive inflammation can delay tissue repair, highlighting the importance of immunomodulation [67]. Numerous studies have demonstrated the immunoregulatory functions of PDNVs, particularly their ability to induce macrophage polarization for tissue repair [68]. For instance, Pueraria lobata-derived nanovesicles have been shown to inhibit M1 pro-inflammatory macrophages and promote the transition to M2 anti-inflammatory macrophages [69]. Similarly, cabbage-derived nanovesicles have been found to reduce the expression level of pro-inflammatory cytokines such as interleukin-6 (IL-6) and cyclooxygenase-2 (COX-2) [57], while garlic chives-derived nanovesicles containing DLPC could impede the activation of the NLRP3 inflammasome [70]. Furthermore, ginger-derived nanovesicles have the capacity to inhibit NLRP3 inflammasome-mediated secretion of IL-1β and IL-18, as well as pyroptosis [33, 71].

In addition to their modulatory effects on macrophages, petasites japonicus-derived nanovesicles could induce dendritic cell maturation through the activation of mitogen-activated protein kinase (MAPK) and nuclear factor-kappa B (NF-κB) pathway, while broccoli-derived nanovesicles could prevent dendritic cells activation by activating the adenosine monophosphate-activated protein kinase (AMPK) pathway [40, 53]. Furthermore, celery-derived nanovesicles have been shown to inhibit the activation of peripheral blood mononuclear cells (PBMC), and aster yomena callus-derived nanovesicles could dampen the immune-stimulating capacity of CD4^+^ and CD8^+^ T cell proliferation and activation, thereby improving various symptoms of asthma in a rat model [66]. Additionally, ginger-derived nanovesicles have been found to mitigate the impact of LPS levels in vivo, suggesting their potential for systematic regulation of inflammation [72]. These findings highlight the diverse immunoregulatory effects of PDNVs. As such, further research is needed to determine the specific selection of plant sources as immune-modulating tools.

### Flora balance

Microbial communities are present in various parts of the human body, such as the gut, oral cavity, and skin, and their balance plays a crucial role in maintaining human health and preventing disease [73]. When there is a disruption in this balance, known as dysbacteriosis, it can lead to the development of various conditions including colitis, inflammatory bowel diseases, skin diseases, and oral inflammation. In this regard, PDNVs have emerged as a promising tool for defending against pathogen invasion and restoring the balance of microbial communities [74].

PDNVs exhibit remarkable abilities in combating pathogens and maintaining microbial balance. They can enter microbes through lipid-dependent uptake and directly eliminate pathogens, thereby exerting antibacterial effects. For example, ginger-derived nanovesicles have been shown to selectively endocytosed by the periodontal pathogen *P. gingivalis*, reducing its virulence through the action of specific lipids (particularly PA (34:2)) and miRNA (such as aly-miR159a). This interaction between nanovesicles and pathogens, facilitated by surface interactions with proteins like hemoglobin-binding protein 35 (HBP 35), helps maintain microbial balance [35]. In addition, Rehmannia-derived nanovesicles could inhibit biofilm formation and target the virulence gene sxt2 through the action of miR-7972 [33]. Similarly, bee pollen-derived nanovesicles have demonstrated significant inhibition of colony and biofilm formation of *Staphylococcus aureus* (*S. aureus*) [75].

PDNVs also show great potential in modulating gut microbiota and promoting tissue health and repair, particularly in the treatment of gastrointestinal diseases. Lemon-derived nanovesicles are capable of downregulating tRNAser^UCC^, which limits the production of methylation-specific restriction endonuclease Msp1 and Msp3 in *lactobacillus rhamnosus GG* (*LGG*). This, in turn, enhances the survival of beneficial gut bacteria [45]. Moreover, ginger-derived nanovesicles could significantly improve the abundance and diversity of gut microbiota. They can reverse the decreased abundance of specific beneficial bacteria, including *Ackermania, Lactobacillus*, *Clostridium_*UCG-014, and *Bifidobacterium,* in a colitis model induced by DSS [76]. These findings hold promise for the alleviation of gastrointestinal tract diseases. However, in practical applications, the ability of PDNVs to specifically regulate the microbiota in individual patients remains to be investigated due to variations in patients' microbial compositions.

### Anti-oxidant activity

Certain diseases can impair the cellular antioxidant capacity, leading to an excessive generation of reactive oxygen species (ROS) and cellular damage [77]. Although nanovesicles derived from plant sources may exhibit lower antioxidant capacity compared to the original juice, their antioxidant effects remain a crucial aspect of their functionality [78, 79]. For example, grapefruit and carrots-derived nanovesicles could reduce ROS production in human immortalized keratinocyte cell lines and H9C2 cardiomyocyte cell lines in a dose-related manner, respectively [21, 80]. Additionally, green tea-derived nanovesicles have been found to significantly upregulate heme oxygenase-1 (HO-1) expression and reduce ROS levels [43]. This effect may primarily depend on the components present in PDNVs. For example, strawberry juice-derived nanovesicles, rich in vitamin C, could effectively reduce ROS production [81].

Nrf2 is a crucial transcription factor involved in the regulation of antioxidant proteins, responsible for transcribing genes such as HO-1, catalase (CAT), and superoxide dismutase (SOD). Several studies have shown that PDNVs could modulate the expression of Nrf2. Blueberries-derived nanovesicles have been shown to alleviate oxidative stress induced by fisetin in hepatocellular carcinoma G2 cells and high-fat diet (HFD)-fed C57BL/6 mice by facilitating the translocation of Nrf2 from the cytoplasm to the nucleus[54]. Similarly, ginger-derived nanovesicles containing shogaol have been found to enhance Nrf2 nuclear translocation in liver cells [44]. However, further investigation is needed to determine the specific components of PDNVs that regulate Nrf2.

PDNVs can also protect cells from oxidative stress caused by external stimuli. Exposure to radiation causes cell damage via excessive production of ROS, with mitochondria being the primary source of ROS within cells. Bitter melon-derived nanovesicles have been shown to mitigate mitochondrial dysfunction in H9C2 cardiomyocyte cell lines following radiation exposure [55]. In vivo experiments have also demonstrated that orally administered mixtures of PDNVs could restore the physiological condition of mice treated with hydrogen peroxide (H_2_O_2_) for 2 weeks, indicating their potent antioxidant capacity and the presence of various bioactive substances, including hydrogen peroxide enzyme, glutathione (GSH), SOD, ascorbic acid, melatonin, phenolic compounds, and adenosine triphosphate (ATP) [82]. These findings highlight the multifaceted role of PDNVs in combating oxidative stress and their potential as therapeutic agents for oxidative stress-related disorders.

### Anti-aging effect

Mitigating cellular aging plays a crucial role in preserving normal cellular function, preventing tissue damage, and promoting tissue repair and regeneration [83]. Ginseng-derived nanovesicles have shown the ability to alleviate replicative senescence in dermal fibroblasts and exhibit anti-aging effects on melanocytes exposed to ultraviolet radiation (UVR)-induced aging, along with anti-pigmentation effects. Treatment with ginseng-derived nanovesicles has been found to significantly and dose-dependently reduce melanin content and senescence-associated β-galactosidase activity in aged cells. Furthermore, the application of 5 μg/ml ginseng-derived nanovesicles has been shown to counteract UVB-induced melanocyte senescence, characterized by flattening and enlargement of cell bodies with an increase in spindle-shaped cells [84]. The prevention of UV-induced aging holds tremendous potential in the beauty industry. However, current research lacks further mechanistic studies and animal experiments to confirm the anti-aging effects.

The biological effects observed in the aforementioned PDNVs form the basis for their tissue protection and repair properties. However, some studies have only conducted a superficial assessment of the PDNVs' activity without delving into the specific underlying mechanisms. It remains unknown whether the components carried by the nanovesicles provide superior effects compared to individual components. Furthermore, it is worth investigating which PDNVs with similar effects exhibit greater efficacy.

## The potential application of PDNVs in tissue protection and repair

### Brain

The brain serves as the command center of our body, and when it experiences damage, it often leads to irreversible consequences. Cerebral ischemia/reperfusion injury is a common type of damage that occurs when the blood supply to the brain is temporarily cut off and then restored, resulting in tissue damage and neurological dysfunction [85]. Current treatments for this condition primarily focus on antioxidant, anti-inflammatory, and neuroprotective strategies. Other potential methods, such as photodynamic therapy, gene therapy, and stem cell therapy, are being explored. However, the effectiveness, safety, and feasibility of these treatments are still not fully satisfactory.

Indeed, the majority of drugs, around 99%, encounter difficulties in traversing the blood–brain barrier to reach brain tissue and exert their therapeutic effects [86]. Fortunately, there is encouraging evidence from multiple studies indicating that PDNVs have the capability to penetrate the blood–brain barrier [23, 24]. Treatment with bitter melon-derived nanovesicles, for example, have been shown to effectively reach the ischemic brain area, strengthen the blood–brain barrier, reduce infarct size, and improve neurofunctional deficit scores in a rat model of cerebral arterial occlusion when administered intravenously. Bitter melon-derived nanovesicles could upregulate the protein kinase B/glycogen synthase kinase-3 beta (AKT/GSK3β) signaling pathway in hippocampal neuronal HT22 cells and reduce neuronal apoptosis. This may be attributed to miR-5266, which decreases the expression of matrix metalloproteinase-9 (MMP-9) while increasing the expression of zonula occludens-1 (ZO-1) and claudin-5, genes that play important roles in maintaining the integrity and function of the blood–brain barrier [87]. The regulation of these genes plays a crucial role in protecting neural tissues.

Systemic diseases or external stimuli can also affect brain tissue, such as brain inflammation caused by obesity or alcohol consumption. PDNVs can also provide beneficial effects in protecting brain tissue from external stimuli. Garlic-derived nanovesicles (GaNVs) could specifically target microglia and suppress brain inflammation in rat obese animal models. Moreover, the metabolites generated by microglia treated with GaNVs have been shown to promote neuronal differentiation and inhibit mitochondrial-mediated neuronal cell death. These findings contribute to enhanced memory function and improved glucose tolerance and insulin sensitivity, indicating that GaNVs hold promise as a potential strategy to alleviate neuroinflammation in mice with diet-induced obesity [72].

**Fig. 3:**
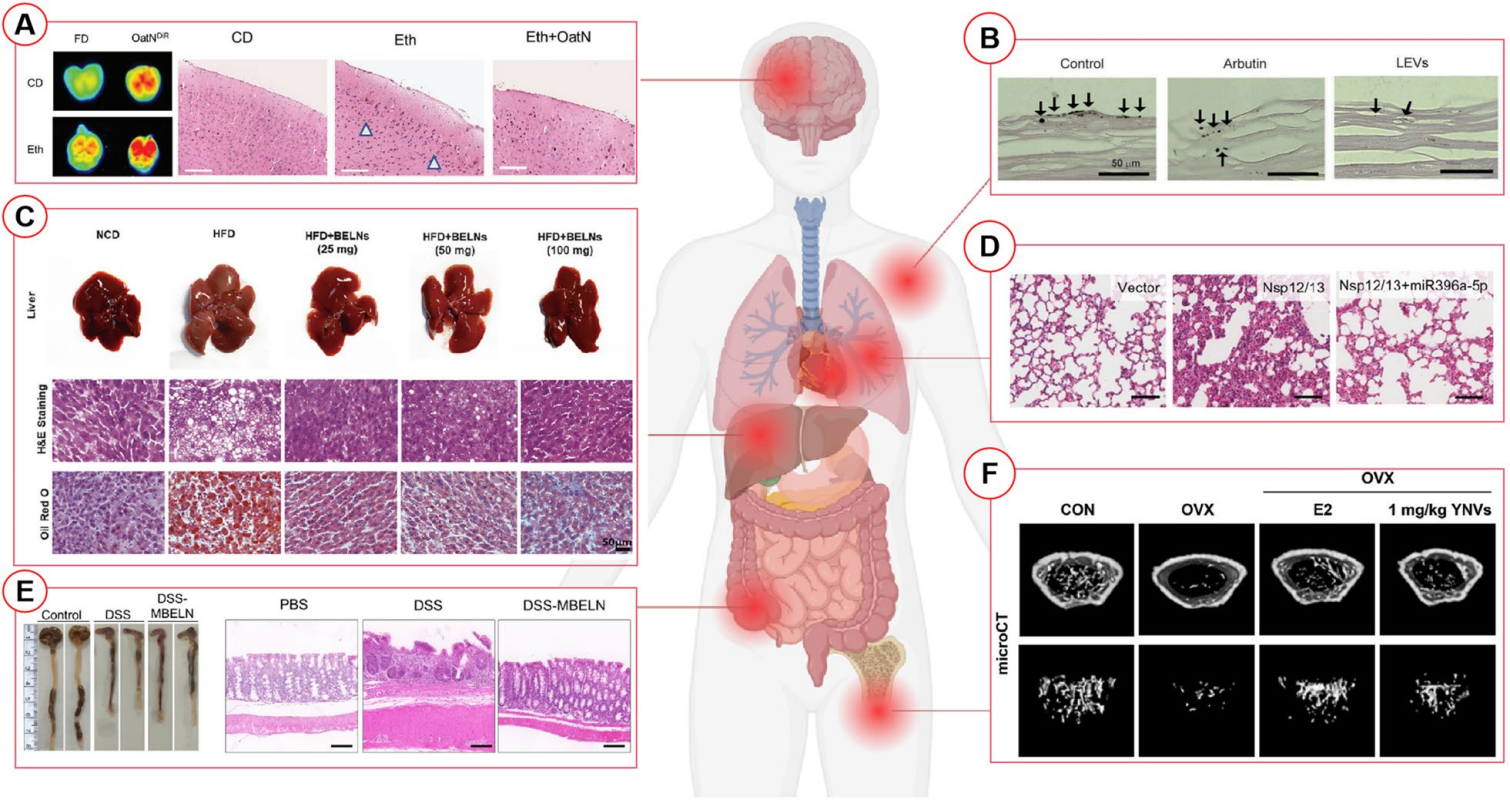
Potential applications of PDNVs in tissue injury repair. **A** Oats-derived nanovesicles could alleviate inflammation in mouse brain tissue caused by ethanol [24]. (*CD* control; *Eth* Ethanol, *OatN* Oat-derived nanovescicles) **B** Dendropanax morbifera-derived nanovesicles could reduce melanin formation in human skin tissue [88]. (LEVs, Dendropanax morbifera leaf-derived extracellular vesicles) **C** Blueberries-derived nanovesicles could alleviate nonalcoholic liver diseases in high-fat diet mouse [54]. (*NCD* normal chow diet, *HFD* high-fat diet, *BELNs*, Blueberries-derived exosomes like nanoparticles) (D) Some key miRNAs in PDNVs could alleviate lung inflammation in a rat model [89]. (Nsp12/13, nonstructural proteins of coronaviruses (including SARS-CoV-2)) **E** Mulberry bark-derived nanovesicles could alleviate mouse colitis induced by DSS [47]. (PBS, phosphate buffered saline, DSS, dextran sodium sulfate; MBELNs, Mulberry bark-derived exosomes like nanoparticles) **F** Yam-derived nanovesicles could enhance bone density in ovariectomy-induced osteoporotic mice [19]. (*CON* control; *YNVs* Yam-derived nanovesicles, *OVX* ovariectomy, *E2* estradiol)

Additionally, ethanol triggers a pathway that induces brain inflammation, but oats-derived nanovesicles could inhibit inflammatory cytokine levels, reduce infiltrating cells in the brain regions (Fig. 3A, as shown by the white triangle in the figure), and enhance memory function in mice fed alcohol [24]. Oats-derived nanovesicles are taken up by microglia through a binding process involving β-glucan and HPCA. Subsequently, DGDG in oats-derived nanovesicles prevents the binding of β-glucan to dectin-1, effectively inhibiting the inflammation pathway. The β-glucan/HPCA complex is then transported to the endosomal recycling compartment (ERC) via Ras-related protein Rab-11a, where it sequesters dectin-1, a key player in immune activation, and modifies its location. This process leads to an increase of dectin-1 into exosomes, ultimately preventing ethanol-induced brain inflammation signaling pathways [24]. However, further applications of PDNVs still require long-term biosafety evaluations and more validation in animal models.

### Skin

The skin, being the largest organ, serves as the primary defense against external factors like ultraviolet radiation, oxidative stress, and physical injuries. However, when exposed to the external environment, the skin is vulnerable to harm. As mentioned earlier, PDNVs have the potential to combat aging when subjected to external ultraviolet radiation. Apples-derived nanovesicles are capable of enhancing collagen synthesis and inhibiting its degradation by suppressing the production of metalloproteinases in dermal fibroblasts. As a result, they effectively alleviate signs of aging [90]. Similarly, ginseng cell culture supernatants or ginseng root-derived nanovesicles could ameliorate replicative senescence or aging-related pigment phenotype in human skin fibroblasts or UVB-irradiated human melanocytes. Extended usage is expected to further enhance their anti-aging effects [39]. Additionally, certain diseases such as diabetes and obesity, can elicit immune responses in the skin. Ginger-derived nanovesicles possess anti-inflammatory properties that can prevent skin damage and the onset of gray hair caused by insulin resistance in a high-fat diet induced mouse [39]. However, the underlying mechanisms responsible for these effects are still not fully understood.

**Fig. 4:**
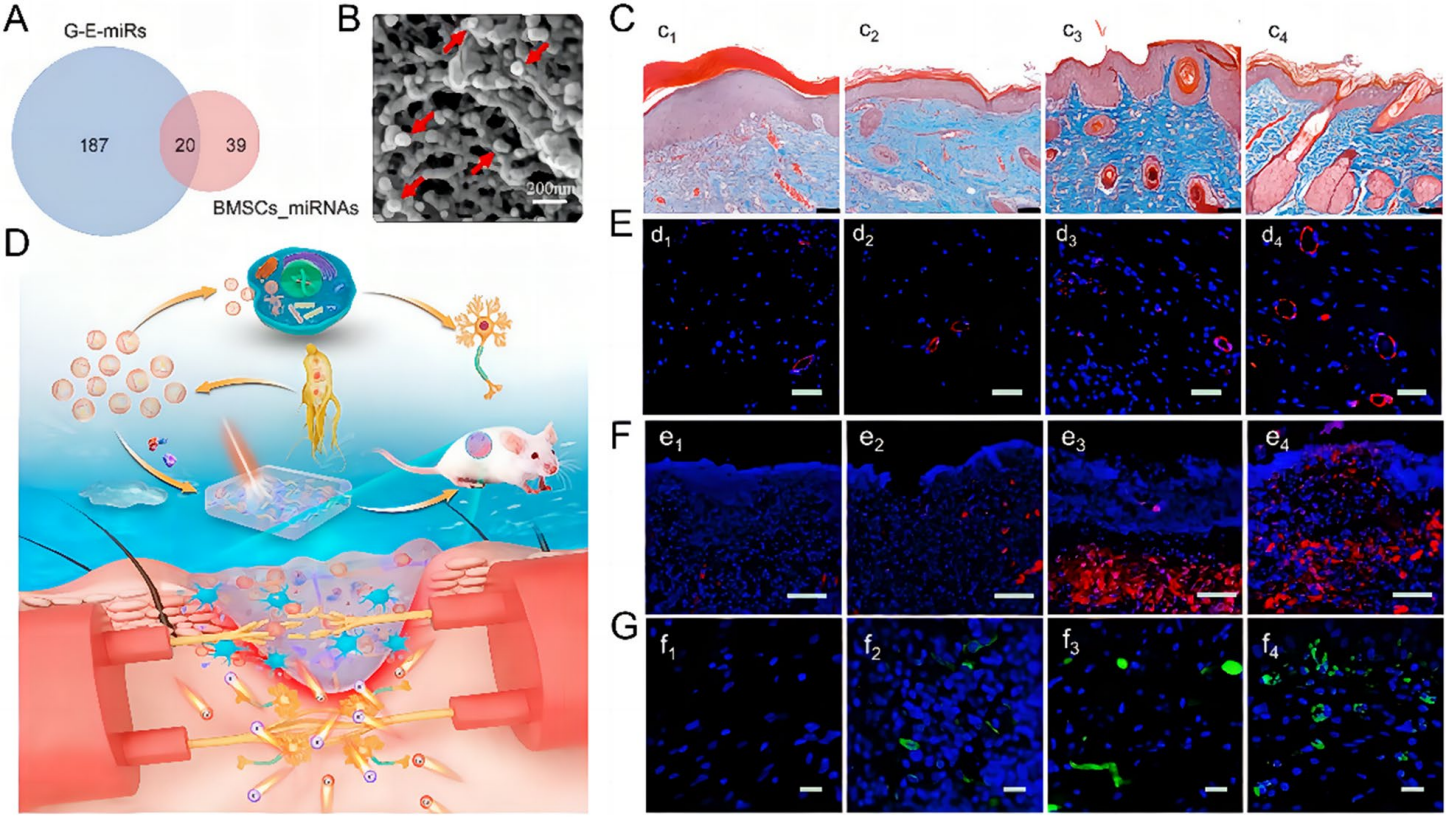
The dressing loaded with ginseng-derived nanovesicles promotes nerve regeneration and blood vessel formation in wounds. **A** The Venn diagram of miRNAs from bone marrow mesenchymal stem cells and G-E. **B** Scanning electron microscopy image of the dressing loaded with G–E (nanovesicles indicated by red arrows). **C** The dressing loaded with G–E promotes regeneration of skin appendages. Expression of **D** CD31 (red, associated with blood vessel formation); **E** CD90 (red, associated with neuronal migration and synaptogenesis); **F** Nestin (green, a marker for neural stem cells and neurodevelopment) in the different dressing group. **G** The wound healing rate among different dressing groups during the healing process. (The lowercase letters c, d, e, f followed by 1, 2, 3, 4 represent the blank group, G-E group, CLD-C group, and CLD-C-E group, respectively.) (**G**–**E**, ginseng-derived nanovesicles; CLD-C-E, CXCL12 and G-Exo-loaded cross-linked gel dressing; CXCL12, C-X-C motif chemokine ligand 12; *CD* cluster of differentiation) (Copyright [20])

While the skin has the inherent ability to heal injuries independently, improving the healing process to reduce scarring and restore sensation function remains a significant challenge. Wheat juice-derived nanovesicles actively promote angiogenesis and facilitate the in vitro proliferation and migration of epithelial and fibroblast cells, which are crucial for wound healing [51]. Furthermore, ginseng-derived nanovesicles not only facilitated the delivery of their miRNA to BMSCs, promoting the neuronal differentiation (Fig. 4A), but also enhance wound healing when synergistically incorporated with C-X-C motif chemokine ligand 12 (CXCL12) into a scaffold (Fig. 4B). These effects are attributed to the stimulation of new tissue and regeneration of skin appendages, including hair follicles and sweat glands in a rat wound model (Fig. 4C). Moreover, they promote the regeneration of blood vessels (Fig. 4D), reduce inflammation [91], and restore sensory perception by facilitating nerve tissue regeneration and neuronal migration (Fig. 4E, F) [20]. From the results, we can observe that this effect requires the synergistic action of chemotactic factors, and this strategy does not significantly enhance the healing efficiency (Fig. 4G). Regardless, it holds great promise for sensory restoration in skin repair, which could be a breakthrough for conditions like diabetic peripheral neuropathy.

In addition to the above effects, it is worth noting that external stimuli, such as ultraviolet radiation, can lead to the accumulation of melanin in the skin, resulting in skin darkening. Skin whitening, undoubtedly, holds significant market potential. PDNVs also demonstrate substantial potential in skin whitening, surpassing even the efficacy of existing strategies in the market. Dendropanax morbifera leaves and stems-derived nanovesicles could reduce melanin content and inhibit tyrosinase (TYR) activity in melanoma cells in a concentration-dependent manner. Their whitening effect might surpass that of the positive control arbutin, significantly reducing the formation of melanin, with leaf-derived nanovesicles exhibiting a stronger impact in a human skin model **(**Fig. 3B, as shown by the black arrow in the figure**)** [88]**.** Similarly, ginseng-derived nanovesicles could reduce melanin levels, surpassing the representative whitening compound melasolv (3,4,5-trimethoxycinnamic acid 4-hydroxyphenethyl ester) at the same protein concentration in human skin cells [84]. These results suggest that PDNVs have the potential to become a rising star in the beauty industry with significant market prospects.

### Heart

Patients undergoing thoracic radiotherapy face the risk of developing radiation-induced heart disease (RIHD) due to the excessive generation of ROS and subsequent oxidative stress [92]. Bitter melon, a plant renowned for its antioxidative properties, has shown promise in safeguarding patients from the detrimental effects of RIHD. Specifically, bitter melon-derived nanovesicles have demonstrated effectiveness in preventing RIHD [55]. These nanovesicles exhibit a time-dependent internalization into rat cardiomyocytes, stimulating cell proliferation while inhibiting apoptosis. Moreover, they diminish deoxyribonucleic acid (DNA) damage and scavenge mitochondrial ROS, thereby preserving mitochondrial function. In irradiated cells, bitter melon-derived nanovesicles also restored the phosphorylation of proteins associated with ROS, further alleviating oxidative stress. In a thoracic mice irradiation model, the administration of bitter melon-derived nanovesicles significantly reduced radiation-induced myocardial injury and fibrosis [55]. These findings suggested that bitter melon-derived nanovesicles hold potential as a therapeutic strategy for preventing RIHD. Their ability to scavenge ROS and restore protein phosphorylation represents a promising mechanism of action. These nanovesicles offer valuable insights for the development of novel treatment options for RIHD, instilling hope for patients undergoing thoracic radiotherapy.

### Liver

The treatment for acute liver injury remains challenging, requiring further investigation into the underlying mechanisms and development of new therapeutic approaches, including those based on natural products, to overcome existing limitations. In a d-Galactosamine and lipopolysaccharide-induced acute liver injury in mice models, administration of mushroom-derived nanovesicles reduced pathological changes, including liver hemorrhage and cell death, while also decreasing inflammation levels and mitigating liver damage [93]. Similarly, local application of chive-derived nanovesicles alleviated NLRP3-mediated inflammation in the same acute liver injury mice model [41]. Cannabis-derived nanovesicles demonstrated the ability to decrease elevated alanine aminotransferase (ALT) and aspartate aminotransferase (AST) levels, reduce liver cell damage, and restore the levels of liver regeneration cell cycle protein D1 and endothelial nitric oxide synthase (eNOS) that were diminished by DSS-induced injury in mice [12]. All of these have significant implications for liver tissue protection.

Treatment strategies for alcohol-induced liver injury include abstinence, medication, nutritional support, and liver transplantation. However, these approaches have limitations and challenges, such as medication side effects and scarcity of liver donors for transplantation. Oat-derived nanovesicles mediate an inhibitory effect on IL-1β, IL-6, and TNF-α expression in the liver following alcohol intake, providing liver protection [24]. Ginger-derived nanovesicles triggered the expression of a group of detoxification/antioxidant genes in the mouse liver, including HO-1, NAD(P)H quinone dehydrogenase 1 (NQO1), glutamate-cysteine ligase modifier subunit (GCLM), and glutamate-cysteine ligase catalytic subunit (GCLC). This leads to lowered liver triglyceride levels, reduced liver weight, and decreased liver lipid droplet accumulation, effectively safeguarding mice from alcohol-induced liver injury. Moreover, shogaol present in ginger-derived nanovesicles could induce Nrf2 nuclear translocation through the toll-like receptor 4/TIR domain-containing adapter-inducing interferon-β (TLR4/TRIF) pathway, activating antioxidants to protect liver cells from oxidative tissue damage [44]. In the presence of therapeutic efficacy demonstrated by many PDNVs, further comparative studies are needed to determine which type of vesicle is likely to exert the optimal hepatoprotective effects on liver tissue.

Non-alcoholic fatty liver disease (NAFLD) is closely associated with mitochondrial dysfunction and oxidative stress [94]. Blueberries-derived nanovesicles could facilitate the translocation of Nrf2 into the nucleus, inhibiting fatty acid synthesis and lipid droplet accumulation **(**Fig. 3C**)**, thereby alleviating oxidative stress and disease progression in a high-fat diet mice model. These nanovesicles could decrease ROS levels in hepatocellular carcinoma G2 cells, enhance mitochondrial membrane potential, and reduce cell apoptosis by regulating the expression of B-cell lymphoma-2 (Bcl-2) and HO-1, while inhibiting the expression of Bcl-2-associated X protein (Bax) [54]. These findings highlight the potential of nanovesicles derived from various natural products as therapeutic strategies for liver diseases. They could offer valuable insights for the development of innovative treatment options, instilling hope for patients grappling with liver diseases.

### Lung

Current treatment approaches for acute lung injury primarily involve mechanical ventilation, fluid management, and nutritional support. However, these methods still present challenges, including the lack of specific therapies and effective prognostic tools [95]. Fresh rehmannia-derived nanovesicles have shown significant anti-inflammatory effects, effectively reducing LPS-induced acute lung injury in mice. The nanovesicles could mitigate the production of pro-inflammatory cytokines, ROS, and nitric oxide (NO), promoting the polarization of anti-inflammatory M2 macrophages. These findings provided innovative perspectives and directions for the research and development of novel therapies for acute lung injury [33]. Furthermore, aster yomena callus-derived nanovesicles have demonstrated potential in alleviating allergic asthma symptoms mediated by ovalbumin in mouse models, possibly by inhibiting the activation and maturation of immune cells induced by LPS [66].

The treatment of Coronavirus Disease 2019 (COVID-19) still faces multiple challenges, including the absence of effective specific drugs and vaccines, as well as uncertainties in treatment protocols [10]. Fortunately, miRNAs in PDNVs hold the potential to target severe acute respiratory syndrome coronavirus 2 (SARS-CoV-2). Kalarikkal et al. predicted that ginger and grapefruit-derived nanovesicles, containing multiple miRNAs, could target different regions of the SARS-CoV-2 genome [96]. Non-structural protein 12 (Nsp 12) and Nsp 13 are crucial components of the SARS-CoV-2 RNA polymerase complex. Indeed, ginger-derived nanovesicles containing aly-miR 396a-5p could inhibit the cytopathic effect triggered by SARS-CoV-2 by suppressing the expression of Nsp 12 and spike genes. This effect holds the potential to reduce pulmonary fibrosis caused by SAR-CoV-2 **(**Fig. 3D**)** [89]. These findings hold paramount significance for the treatment and prevention of COVID-19, offering potential methodologies and strategies for the development of new treatments targeting SARS-CoV-2.

### Gastrointestinal tract

Functional disorders, inflammation, infections, or injuries affecting the digestive system can result in indigestion, pain, ulcers, bleeding, and even cancer. However, there are still challenges in terms of limited treatment options, drug resistance, and recurrence of chronic disease. Restoring the equilibrium of the gut microbiota plays a vital role in protecting and repairing gastrointestinal tissues. In this regard, PDNVs offer a safe and natural alternative for modulating the gut microbiota. For example, a noticeable decrease in the *Firmicutes*/*Bacteroidetes* ratio may indicate remission in inflammatory bowel disease. Administration of tea-derived nanovesicles have been shown to significantly reduce the *Firmicutes*/*Bacteroidetes* ratio and increase the abundance of beneficial bacteria like *Bifidobacterium* and *Trichophytonaceae* [43]. Moreover, tea-derived nanovesicles exhibit potential in preventing colitis-related cancer, while tea flowers-derived nanovesicles may aid in regulating gut microbiota equilibrium and reducing tumor metastasis [43, 97].

Colitis stands as one of the most prevalent gastrointestinal tract diseases, and the standard treatment primarily relies on immunosuppressants and anti-inflammatory drugs, which come with their own limitations and side effects [98]. PDNVs show great promise in colitis treatment, given that most food is ingested and reaches the intestine. In a mouse model of DSS-induced colitis, Sriwastva et al., found that compared to the PBS-treated or control group, colon length was significantly reduced after treatment with mulberry bark-derived nanovesicles, and the damage to crypts was markedly attenuated (Fig. 3E) [47]. In addition, administration of ginger-derived nanovesicles resulted in significantly decreased mortality rates compared to untreated mice, while also exhibiting comparable villus height, increased numbers of intestinal stem cells, and the promotion of β-catenin nuclear translocation in the intestinal epithelium [12]. Different parts of the hemp (root, leaf, flower, and seed)-derived nanovesicles have shown the ability to restore tight junction/adherens junction proteins, decrease NF-κB activation, and reduce markers of oxidative stress [12]. These food-derived nanovesicles naturally occur in food and if their oral administration can provide protective and reparative effects on the gastrointestinal tract, it holds promising prospects. However, the specific mechanisms of action and long-term biological safety still require evaluation.

Furthermore, gastrointestinal complications can also arise due to obesity and other systemic burdens. Oranges-derived nanovesicles have been found to increase villus size in the duodenum, lower triglyceride levels, and regulate mRNA levels of genes involved in immune response, barrier permeability, fat absorption, and chylomicron release. These nanovesicles target microsomal triglyceride transfer protein (MTP) and angiopoietin-like protein-4 (ANGPTL 4) in the mitochondria, which are therapeutic targets for reducing plasma lipids and inflammation in gastrointestinal diseases caused by obesity [99]. The utilization of PDNVs as a strategy to protect gastrointestinal tissues from damage and promote healing holds great potential for application, especially considering their abundant presence in our daily diet. Many PDNVs have demonstrated properties such as gut microbiota modulation and promotion of gastrointestinal diseases repair. It is crucial to further investigate their efficacy and elucidate the underlying mechanisms to facilitate their future applications.

### Bone

Osteoporosis is a chronic condition characterized by reduced bone mass and fragile bones, increasing the risk of fractures. Current treatments for osteoporosis include dietary modifications, exercise, medications, and surgery. However, these treatment strategies have limitations and challenges, such as side effects and surgical complications. Encouragingly, apple-derived nanovesicles have shown the ability to promote the osteogenic differentiation of MT3T3 osteoblasts in vitro [50]. Similarly, yam-derived nanovesicles have demonstrated the potential to enhance bone regeneration, bone volume density, and bone formation rate in vivo. At a dosage of 1 mg/kg, yam-derived nanovesicles exhibited effects of increasing bone density in a mouse model of ovariectomized-induced osteoporosis comparable to those of estradiol (E2), a hormone used for osteoporosis (Fig. 3F). Compared to the numerous side effects associated with hormone therapy, yam-derived nanovesicles hold promise for exhibiting a milder effect and possessing some degree of bone tissue targeting. Their study also confirmed the favorable organ and blood biocompatibility of these nanovesicles [19]. Nevertheless, further investigation is needed to elucidate the underlying mechanisms of action and to evaluate their long-term safety and efficacy in treating osteoporosis.

Periodontitis, a prevalent disease affecting the alveolar bone, is triggered by dental plaque, particularly *P. gingivalis*, leading to inflammation and destruction of the supportive tissues surrounding the teeth [100]. Current treatments for periodontitis include mechanical debridement and pharmacological therapy. However, they face challenges such as drug resistance, adverse reactions, and limited efficacy for intricate lesions. The PA (34:2) present in ginger-derived nanovesicles can specifically bind to the HBP 35 in *P. gingivalis*, mitigating its pathogenicity and reducing its adherence to gingival epithelial cells. Administration of ginger-derived nanovesicles in drinking water has been shown to decrease gingival inflammation and significantly increase alveolar bone density in a mouse periodontitis model [35]. These findings suggested that PDNVs have the potential to be a promising therapeutic approach for periodontitis, offering additional options and optimism for managing this disease.

The examples mentioned above have demonstrated the potential applications of PDNVs in tissue protection and repair (Fig. 3) (Table 2). PDNVs offer several advantages in this field, including, but not limited to: (1) Some PDNVs exhibit natural targeting ability, as not all PDNVs will migrate to bone tissue after intravenous injection [19]; (2) PDNVs can traverse the blood–brain barrier, providing a potential treatment option for intracranial diseases [23, 24]. (3) PDNVs possess excellent biocompatibility, particularly certain nanovesicles derived from food sources that exhibit microbial modulation effects in gastrointestinal diseases [43]. (4) PDNVs can serve as a "protective umbrella" for preventing organ damage caused by various diseases and external stimuli, such as alcohol [24], ultraviolet radiation [39], and obesity [39].

**Table 2:** The potential of PDNVs facilitate tissue protection and repair in vivo

Organ/Tissue	Plant sources	Administration method; dosing interval	Effects	Refs.
Skin	Dendropanax morbifera	Smeared on the skin: 10 μg/mL for 7 days	In vitro: It reduced the expression of MITF, TYR, TRP-1 and TRP-2, resulting in a reduction in cellular melanin synthesis In vivo: It exerted a stronger inhibitory effect on melanin production	[88]
	Ginseng	Smeared on the skin: 20 μL of GDNPs (10 mg/mL), every 24 h for 15 days	In vitro: It regulated HUVECs and HaCaT cells proliferation via ERK and AKT/mTOR pathways In vivo: It facilitated skin wound healing and decreased inflammation	[91]
	Ginseng	The defect sites were filled with 200 μL of CXCL12 and Ginseng Exosome-loaded cross-linked gel dressing	In vitro: It regulated the PI3K signaling and induced neural differentiation in BMSCs In vivo: It induced neural restoration	[20]
Bone	Yam	Oral administration: 1 mg/kg, 3 days per week	In vitro: It activated the BMP-2/p-p38-dependent Runx2 pathway to osteogenesis In vivo: It promoted longitudinal bone growth and mineral density in the tibia of osteoporotic mice	[19]
	Ginger	Oral administration: 4.0 × 10^8^ particles/mL in drinking water	In vitro: It inhibited the growth of *P.gingivalis* by directly interacting with the HBP35 protein In vivo: It affected the growth, attachment, entry, and proliferation of host cells, resulting in a reduction of pathogenic virulence	[35]
Brain	Momordica charantia	Intravenous injection: 200, 400, or 800 μg/kg	In vitro: It activated the AKT/GSK22β signaling pathway in HT3 cells and attenuated neuronal apoptosis In vivo: In brain I/R injury, it down-regulated MMP-9 expression and up-regulated the expression of tight junction proteins claudin-5 and ZO-1, significantly reducing infarct size and mNSS score, and protecting BBB integrity	[87]
	Oat	Oral administration: 8 mg/kg of body weight 3 times per week	In vitro: It regulated the coordinated assembly of the HPCA/Rab11a/Dectin-1 complex In vivo: It helped prevent alcohol-induced brain inflammation	[24]
	Garlic	Oral administration: 10^10^ particles, every day for 6 weeks	In vitro: It bound to BASP1 and inhibited c-Myc expression and activity by competitively binding CaM with c-Myc transcription factor. Inhibition of STING activity reduces the expression of a range of inflammatory cytokines, while promoting neuronal differentiation and inhibits mitochondria-mediated neuronal cell death In vivo: It improved memory function, glucose tolerance and insulin sensitivity	[72]
Heart	Momordica. charantia	Intraperitoneal administration: 100 μg/kg, every other day for 5 times	In vitro: It suppressed cell apoptosis to alleviate the DNA damage and elevated mitochondria ROS to rebalance mitochondria membrane potential in irradiated H9C2 cells In vivo: It mitigated myocardial injury and fibrosis	[55]
Liver	Shiitake mushroom	Intraperitoneal administration: 1 × 10^10^ particles/g	In vitro: It inhibited NLRP3 inflammasome activation by preventing inflammasome formation in primary macrophages In vivo: It protected mice from fulminant hepatic failure	[93]
	Garlic chive	Intraperitoneal administration:1 × 10^10^ particles/g	In vitro: It inhibited pathways downstream of NLRP3 inflammasome activation in primary macrophages In vivo: It accumulated in specific tissues and suppressed activation of the NLRP3 inflammasome and chronic inflammation	[41]
	Blueberry	Intragastric administration: 25, 50, or 100 mg/kg, once every other day for a continued 4 weeks	In vitro: It decreased the level of ROS by inducing the expression of Bcl-2 and HO-1 and decreasing the content of Bax in rotenone-treated HepG2 cells In vivo: It improved insulin resistance, ameliorated the dysfunction of hepatocytes, and prevented the formation of vacuoles and attenuated the accumulation of lipid droplets by inhibiting the expression of fatty acid synthase	[54]
	Ginger	Oral administration:50 mg/mouse/day for 7 days	In vitro: It mediated activation of Nrf2 led to the expression of a group of detoxifying/antioxidant genes and inhibited the production of ROS In vivo: It partially contributed to the liver protection	[44]
Gastrointestinal tract	Peeled Hawaiian ginger roots	Mouse: oral gavaged at 500 mg/kg. Human: oral administration: 200 mg in 10 ml of sterile 0.9% sodium chloride every other day for 6 days	In vivo: It could ameliorate colitis via IL-22-dependent mechanisms	[25]
	Mulberry bark	Oral administration: 10 × 10^9^ particles/100 μL/mouse once every day	In vitro: It promoted heat shock protein family A (Hsp70) member 8 (HSPA8)‐mediated activation of the AhR signaling pathway in intestinal epithelial cells In vivo: It conferred protection against colitis	[47]
	Rehmannia	Oral administration: 0.8 mL of herbal juice (contain 2.6 × 10^7^particles)	In vitro: It alleviated LPS-induced lung inflammation by targeting the GPR161-mediated Hedgehog pathway In vivo: It recovered gut microbiota dysbiosis	[33]
	Grape	Oral administration: 2 mg/mouse/day	In vitro: It caused significant induction of Lgr5^hi^ intestinal stem cells through the Wnt/β-catenin pathway In vivo: It could penetrate the intestinal mucus barrier, leads to protection of mice from dextran sulfate sodium-induced colitis	[37]
	Ginger	Oral administration: 0.3 mg/mouse/day for the duration of the study	In vitro: It increased the survival and proliferation of IECs, In vivo: It reduced the pro-inflammatory cytokines and increased the anti-inflammatory cytokines to attenuate damaging factors while promoting the healing effect	[125]
	Hemp	Oral administration: 1 mg/kg/day for 7 days	In vitro: It restored the tight (ZO-1, claudin-4, occludin) and adherent junctions (E-cadherin and α-tubulin) and reduced oxidative stress proteins of NF-κB activation In vivo: It assisted in the recovery from dextran sulfate sodium-induced injury in the small intestine and colon	[12]
	Turmeric	Oral administration: 3 mg/dose/day for 7 days	In vitro: It regulated the expression of the pro-inflammatory cytokines via NF-κB pathway In vivo: It could ameliorate colitis and accelerate colitis resolution	[70]
	Broccoli	Oral administration: 250 μg/mouse/day for 10 days	In vitro: It enhanced AMPK signaling and might promote induction of AMPK-activated anti-inflammatory factors In vivo: It ameliorated murine colitis by inducing regulatory DCs	[40]
	Aloe	Oral administration: 1 mg/kg for 7 days	In vitro: It restored TJ and AJ proteins and played a role in anti-inflammatory and antioxidant properties In vivo: It reduced DSS-induced colonic inflammation to prevent gut permeability	[126]
	Tea leaves	Oral administration: 2 mg/kg per mouse for 5 days	In vitro: It was able to reduce the production of reactive oxygen species, inhibit the expression of pro-inflammatory cytokines, and increase the amount of anti-inflammatory IL-10 secreted by macrophages In vivo: It could efficiently inhibit the inflammatory bowel responses, restore disrupted colonic barriers and enhance the diversity and overall abundance of gut microbiota, thereby preventing or alleviating inflammatory bowel disease	[43]
	Ginger	Oral administration: 6 × 10^8^ particles /mL in the drinking water for at least 12 months	In vitro: By increasing the expression of Foxa2 and protecting it against Akt-1 mediated phosphorylation and subsequent inactivation, it altered the composition of intestinal epithelial cells In vivo: It prevented insulin resistance and inhibited skin inflammation	[39]
	Orange juice	Gavaged administration: 150 μg in 100 μL of PBS, 5 days/week for 4 weeks	In vitro: It induced the release of Chylomicron-Associated triglycerides In vivo: It modulated jejunum morphology and mRNA levels of genes involved in permeability	[99]
	Grapefruit	Oral administration: 10 mg/kg/day for 7 days	In vitro: It induced the expression of the antioxidant gene HO-1 and suppressed the production of proinflammatory cytokines in intestinal macrophages In vivo: It significantly reduced the severity of dextran sulfate sodium-induced colitis	[103]
	Ginger, carrots, grapes, grapefruit	Gavaged administration	In vitro: It promotes interspecies communication by activating genes that regulate anti-inflammatory cytokines, antioxidation, and Wnt signaling In vivo: It is crucial for maintaining intestinal homeostasis	[127]
	Grape	Gavaged administration: 0.5 mg/Rat in 1 mL PBS daily for 2 weeks	In vitro: It enhanced the expression of LGR5 gene of the intestinal stem cells In vivo: It modulated the stem cell microenvironment for intestinal remodeling	[128]
Lung	Ginger	Intratracheal injection: 5 × 10^8^ particles/kg were dispensed into the lung in a single fluid motion	In vitro: It inhibited the SRAS-CoV-2 cytopathic effect in Vero E6 cells by inhibiting the expression of the viral S and Nsp12 In vivo: It contributed to the development of lung inflammation	[89]
	Aster yomena Callus	Oral administration: 4 and 8 mg/kg/day for 6 days	In vitro: It inhibited the phenotypic and functional maturation of LPS-treated dendritic cells, resulting in decreased immunostimulatory capacity during the induction of CD4 and CD8 T cell proliferation and activation. In vivo: It inhibited T cell reactions associated with the etiology of asthma and improved various symptoms of asthma	[66]

*MITF* melanogenesis associated transcription factor, *TYR* tyrosinase, *TRP* tyrosinase related protein*, GDNPs* ginseng-derived nanoparticles, *HUVECs* human umbilical vein endothelial cells, *HaCaT* human deratinocytes cells, *ERK* extracellular regulated protein kinases, *AKT* AKT Serine/Threonine Kinase, *Mtor* protein kinase B, *CXCL12* C-X-C motif chemokine ligand 12, *PI3K* the phosphatidylinositol3-kinase, *BMSC* Bone marrow mesenchymal stem cell; *BMP-2* Bone morphogenetic protein-2; *P38* P38 mitogen-activated protein kinases; *Runx2* runt-related transcription factor 2; *HBP35* hemin-binding protein 35, *GSK3β* Glycogen Synthase Kinase 3 Beta, *I/R* *injury* Iischemia/reperfusion injury, *ZO-1* zonula occludens-1, *mNSS score* modified neurological severity score; *BBB* blood–brain barrier; *HPCA* hippocampal calcium-binding protein; *Rab11A* ras-related protein rab-11A, *BASP1* brain acid soluble protein 1; *CaM* calmodulin; *STING* stimulator of interferon genes; *ROS* reactive oxygen species, *NLRP3* the NOD-like receptor family pyrin domain-containing protein 3; *Bcl-2* B-cell lymphoma-2; *HO-1* heme oxygenase-1; *Bax* bcl-2 assaciated x protein, *Nrf2* nuclear factor erythroid 2-related factor 2; *IL-22* interleukin-22, *HSPA8* heat shock protein family A (Hsp70) member 8; *AhR* the aryl hydrocarbon receptor, *GPR161* G-protein coupled receptor 161; *LPS* lipopolysaccharide; *Lgr5* leucine-rich repeat-containing g-protein coupled receptor 5; *DSS* dextran sulfate sodium; *IECs* intestinal epithelium cell; *NF-κB* nuclear factor kappa-B; *AMPK* adenosine 5′-monophosphate-activated protein kinase, *TJ and AJ* proteins, tight and adherens junction proteins, *IL-10* interleukin-10; *Foxa2* Forkhead box protein A2; *Nsp12* the non-structural protein 12 of coronaviruses

## Strategies to amplify the tissue protection and repair efficiency of PDNVs

As discussed earlier, PDNVs possess inherent properties that promote tissue repair and protection, such as cell proliferation, differentiation, and resistance to external stimuli. These characteristics make them promising for tissue and organ protection and facilitating injury repair. Given their vesicular structure, PDNVs have the potential to serve as effective platforms for drug delivery. Loading therapeutic agents can significantly enhance their therapeutic benefits. Exploiting the unique characteristics of different organs and tissues to inspire surface modifications of PDNVs holds promise for targeted delivery. The administration route of PDNVs also affects their distribution within the body, highlighting the importance of selecting appropriate delivery methods. By implementing proper modifications, drug loading, and suitable administration approaches, the activities of PDNVs can be enhanced.

### Surface functionalization

#### Enhancing circulatory stability

To ensure a larger number of PDNVs reach specific organs, it is crucial to extend their circulation stability. Factors such as delivery methods can influence this stability. For example, oral administration significantly improves the in vivo bioavailability of tea tree flower-derived nanovesicles compared to intravenous injection [97]. Modifications of nanovesicles could also play a role. For instance, post-incubation with polyethylene glycol (PEG) preparations, aloe vera-derived nanovesicles demonstrated significantly improved blood circulatory stability [101]. Similarly, grapefruit-derived nanovesicles modified with heparin exhibited resistance to complement activation, thereby enhancing their in vivo bioavailability. This increased stability allowed the nanovesicles to penetrate the blood–brain barrier and accumulate within intracranial gliomas [23].

#### Enhancing therapeutic efficacy

**Fig. 5:**
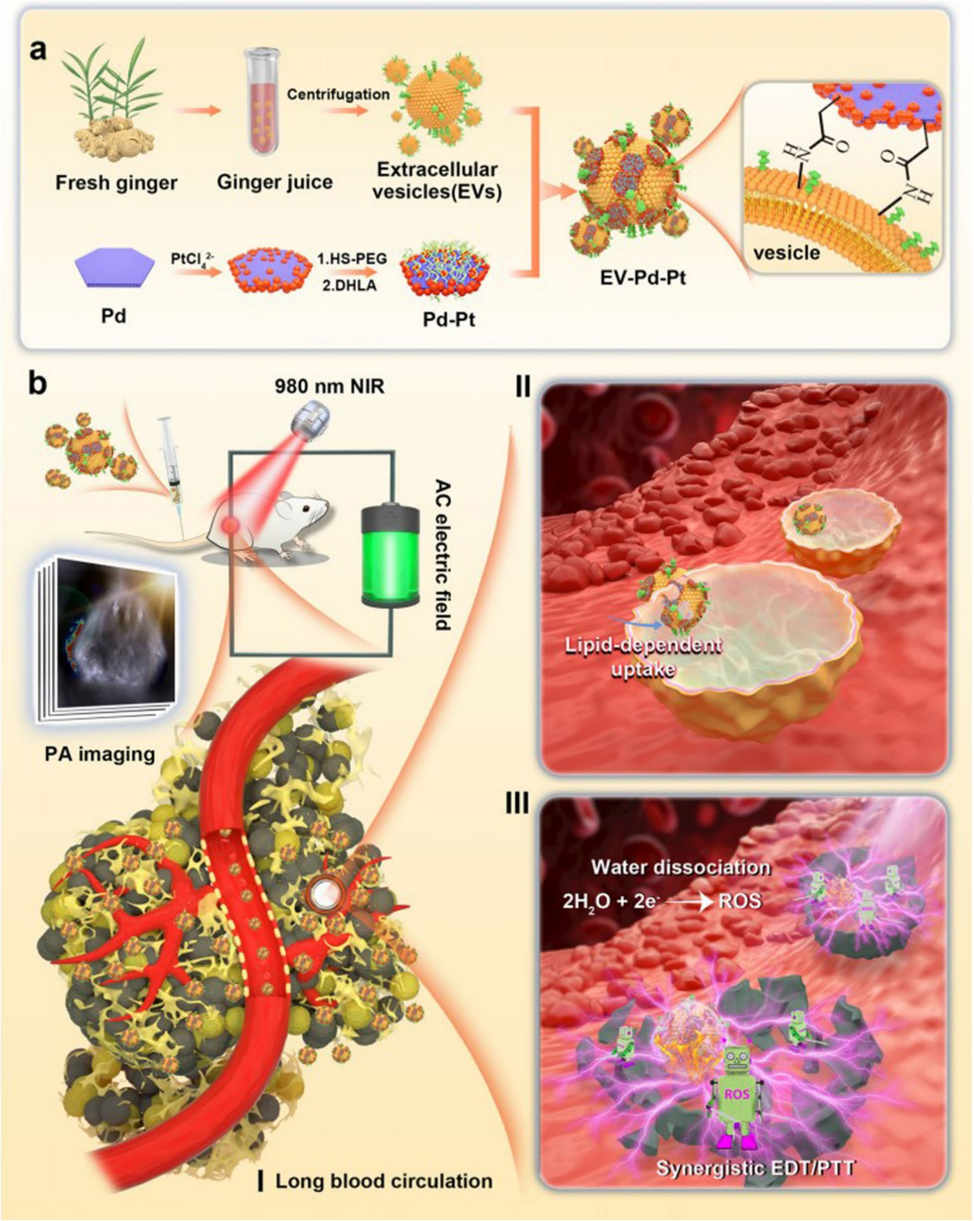
Engineered ginger-derived nanovesicles facilitate synergistic anti-infective therapy [38]. **a**. Extracellular vesicles (EVs) were obtained from fresh ginger juice through centrifugation, and Pt was conjugated onto Pb, followed by their covalent linkage through amide bonds to form EV-Pb-Pt. **b**. Intravenous injection of EV-Pb-Pt exhibited several advantages: I. Prolonged blood circulation stability; II. Lipid-dependent uptake; III. Synergistic effects of an electric field and near-infrared (NIR) light at 980 nm, resulting in the generation of reactive oxygen species (ROS) and exhibiting antibacterial effects. (*NIR* near-Infrared, *ROS* reactive oxygen species, *PA* photoacoustic, *AC* alternating current) (Copyright [38])

Modifying PDNVs is a highly promising strategy for significantly enhancing therapeutic efficacy. Researchers have successfully developed Pd–Pt nanosheets that possess both electrocatalytic and photothermal capabilities. When co-incubated with ginger-derived nanovesicles, the activated carboxyl groups on the nanosheets formed amide bonds with the surface amino groups of nanovesicles (Fig. 5a). The remarkable biocompatibility of ginger-derived nanovesicles enabled prolonged circulation stability in the body (Fig. 5b–I) and accumulation at infection sites. Simultaneously, these nanovesicles could be taken up by bacteria through lipid-dependent mechanisms (Fig. 5b–II). Through the synergistic effects of electric field and photothermal properties, the Pd–Pt nanosheets could continually generate ROS within the bacteria, leading to robust anti-infection outcomes (Fig. 5b–III) [38]. Certainly, PDNVs have been demonstrated to synergistically enhance therapeutic effects when co-administered with various drugs.

#### Enhancing targeted effects

Targeted modification of PDNVs represents a promising strategy for improving their therapeutic efficacy. Current research primarily focuses on anti-tumor applications, employing techniques such as co-incubation to attach antibodies that specifically target certain cells on the surfaces of PDNVs. For instance, in one study, white blood cell membranes were encapsulated on grapefruit-derived nanovesicles, resulting in increased accumulation in inflammatory tissues [102]. In another investigation, arginine-glycine-aspartic acid (RGD) peptides were loaded onto the surface of nanovesicles, significantly enhancing their ability to penetrate brain tissue and target gliomas. These modifications enable PDNVs to more effectively target specific cells or tissues, thereby augmenting their therapeutic efficacy [23]. However, the application of such modifications in organ injury repair remains limited, underscoring the need for further exploration in this area.

### Encapsulation of drugs

Compared to traditional drug therapies, nanomedicine delivery systems have demonstrated numerous advantages such as drug protection, targeting and stability, enhanced drug solubility, crossing biological barriers, and reduced dosage and toxicity. In contrast to conventional nanomedicine delivery systems, PDNVs possess several unique advantages as natural drug delivery systems. These advantages include, but are not limited to: (1) Natural drug carriers: Some drugs naturally exist within PDNVs, serving as innate drug carriers [18]. Concurrent drug administration holds the potential to enhance therapeutic effects. (2) Natural drug delivery system: Due to their natural lipid bilayer vesicle-like structure, PDNVs can serve as natural drug delivery tools without requiring elaborate preparation processes. (3) Natural targeting: PDNVs exhibit inherent targeting potential due to the differential molecular enrichment in their lipid bilayer. For instance, loading methotrexate into grapefruit-derived nanovesicles can selectively target intestinal lamina propria macrophages [103]. (4) Excellent biocompatibility: PDNVs possess favorable biocompatibility, causing minimal immune reactions or toxic side effects. Additionally, when intravenously administered to pregnant mice, they do not cross the placental barrier [104]. (5) Sustainability and environmental friendliness: Plants are commonly renewable resources, and their extraction process is relatively simple and sustainable. Several reviews have summarized the advantages of PDNVs as drug delivery tools [28, 105], we focus on the effects of PDNVs as drug delivery tools in tissue protection and repair.

#### Nucleic acid

Numerous PDNVs exhibit efficient loading and promising therapeutic efficacy for nucleic acid drugs. In one study, miRNA loaded onto cabbage-derived nanovesicles increased miRNA levels by 667,000 times. After co-cultivation with colon cancer cells for 72 h, miRNA levels were observed to increase over 246,000 times in the cells treated with cabbage-derived nanovesicles [57]. Acerola-derived nanovesicles could also successfully deliver nucleic acid drugs to the digestive system through oral administration [106]. Ginger-derived nanovesicles carrying siRNA-CD98, when orally administrated, could effectively and specifically target colon tissue, reducing CD98 expression. This holds the potential for enhancing the therapeutic efficacy of these nanovesicles in ulcerative colitis [107].

#### Compounds

Garlic chives-derived nanovesicles exhibited inherent anti-neuroinflammatory properties. By encapsulating the anti-inflammatory drug dexamethasone into these nanovesicles, inflammation in microglia cells could be further alleviated [108]. Another study involved loading astaxanthin into poly (lactic-co-glycolic acid) (PLGA) nanoparticles, which were then encapsulated into broccoli-derived nanovesicles. This approach significantly enhanced the bioavailability and therapeutic efficacy of the compound [109]. In addition, the side effects of chemotherapy drugs on organs such as the heart should not be overlooked. It has been demonstrated that encapsulating the chemotherapy drug Dox into nanovesicles derived from Beta vulgaris can significantly reduce the toxicity of chemotherapy drugs on organs such as the heart. This has significant potential for application [110].

When utilizing PDNVs as drug carriers, it's crucial to consider the method of drug loading into nanovesicles. Studies have demonstrated that drugs can be loaded into nanovesicles through various techniques such as electroporation, co-extrusion, co-incubation, and repeated freeze–thaw methods. For instance, one study attempted to load curcumin into tomato-derived nanovesicles using different methods (co-incubation, sonication, and extrusion), and found co-incubation yielded the most efficient loading rate of 15.36% [111]. Although this efficiency is suboptimal, strategies used for liposomes as drug delivery systems could provide inspiration for improvements. The ‘‘Sonication and Extrusion-assisted Active Loading (SEAL)’’ method, for example, has been employed to load compounds into milk-derived extracellular vesicles, significantly enhancing drug loading efficiency [112]. Both Zhang et al. and Zeng et al. discovered that the ratio of nanovesicles to drugs affects drug encapsulation efficiency [27, 113]. In their studies, when the concentration ratio of nanovesicles to dexamethasone was 1:2 or even 1:4 using electroporation, the drug loading efficiency was reached 70% to 80% [113].

### Optimization of administration

The choice administration route plays a critical role in the in vivo distribution of PDNVs, which directly impacts their effectiveness **(**Table 3**)**. The primary administration routes include oral administration, intravenous administration, and intraperitoneal administration. Oral administration is highly convenient, safe, and cost-effective. PDNVs can be directly added to food, water, or incorporated into capsules, although the utilization of capsules in this field is not yet common [114]. When employing this administration route, it is important to consider factors such as enzymatic degradation in the oral cavity and the ability to withstand harsh environments like gastric acid or bile [97, 115].

**Table 3:** Advantages, disadvantages, and potential distribution of different administration routes

Methods	Advantages	Disadvantages	Potential distribution
Oral administration	Convenient, economical, easy to operate	May be influenced by oral enzymes and gastrointestinal microenvironment, metabolized by the liver	Digestive system
Transdermal delivery	PDNVs have skin permeability, economical, easy to operate, avoid first-pass elimination	Requires carriers such as patches, susceptible to influences like sweat	Skin tissue
Scaffold loading	Local sustained release	Potential side effects from the use of scaffold	Localized tissue damage
Intraperitoneal injection	Fast absorption, high bioavailability	Invasive	Mainly in immune organs
Intravenous injection	High bioavailability	Invasive, high level of difficulty in operation	Lungs and liver
Intranasal administration	Fast absorption, convenient, easy to operate	Drug limitations, potential nasal irritation, possible passage into the throat	Lungs and brain
Intramuscular injection	Fast absorption, relatively easy to operate	Slow absorption rate, potential for pain and discomfort	Muscular

On the other hand, intraperitoneal administration of chrysanthemum and celery-derived nanovesicles resulted in extensive distribution in immune organs [116, 117]. Macrophages primarily absorb the vesicles following intraperitoneal administration, as demonstrated by the significant reduction of vesicle signals in the spleen after macrophage elimination [68]. Furthermore, after intravenous administration, PDNVs predominantly accumulate in the liver and spleen, which are parts of the mononuclear phagocytic systems (MPS). This accumulation is likely facilitated by the high expression of scavenger receptors on macrophage surfaces [101]. The distributions were also influenced by the composition and size of the vesicles. For example, Zhang et al. discovered that grape and ginger-derived nanovesicles, when administered orally, targeted the liver and intestine, respectively, possibly due to their distinct contents of certain compounds, such as PA and PC, which conferred them with specific ‘‘natural targeting’’ properties.

Moreover, PDNVs possess the ability to penetrate skin tissue and can exert their effects through simple topical application, making them highly advantageous as a potential alternative treatment for skin-related disorders [27, 91]. In cases of local tissue damage, such as skin defects, loading PDNVs into scaffolds (Fig. 4B) enables local sustained release and promotes wound healing capabilities [20]. PDNVs also exhibit excellent characteristics for crossing the blood–brain barrier. For example, in a cerebral ischemia model, bitter melon-derived nanovesicles were found to effectively penetrate brain tissue via the blood–brain barrier following tail vein injection [87]. Notably, intranasal delivery is a viable option for delivering nanovesicles to the brain. Wang et al. found that after intranasal administration, grapefruit-derived nanovesicles were primarily localized in the lungs and brain, while they were mainly found in muscles after intramuscular injection [104]. Intramuscular administration is particularly suitable for vaccine development. Pomatto et al. utilized PDNVs as carriers for SARS-CoV-2 S1 mRNA and observed that intramuscular administration triggered the production of specific antibodies, with IgA serving as the mucosal barrier in the adaptive immune response [118]. Therefore, to achieve satisfactory therapeutic outcomes in vivo, it is crucial to consider suitable administration routes that enhance the accumulation of PDNVs in the target organs.

## Predicting the possible effects of PDNVs

The global existence of an estimated 300,000 to 400,000 plant species highlights their importance as resources for human survival. With a growing interest in discovering novel medicinal properties from plants, researchers have turned their attention to exploring the characteristics of PDNVs in recent years [119]. These nanovesicles, encapsulating RNA, lipids, proteins, and various compounds, exhibit antibacterial, anticancer, antioxidant, and anti-inflammatory effects, thereby holding potential advantages in cancer therapy, tissue protection, and repair. However, the identification of PDNVs with desired activities requires extensive research.

Numerous plants traditionally used in medicine demonstrate antioxidant, anti-inflammatory, and anti-cancer properties, primarily due to the presence of bioactive compounds [120]. Nonetheless, NVs derived from different plants may not necessarily exhibit similar activities as their parent plants. For example, aside from ginger-derived nanovesicles, the inhibitory effects of nanovesicles derived from eight fruits and vegetables (coriander, aloe vera, grapefruit, garlic, turmeric, dandelion, lavender, and cactus) on NLRP3 inflammasomes activation in primary macrophages were minimal compared to their original plant sources [71]. On one hand, PDNVs do not inherit all the bioactive substances from their parent plants. For instance, yam-derived nanovesicles with osteogenic activity, while lacked diosgenin and dioscin [19]. Moreover, catharanthus roseus-derived nanovesicles do not contain well-known compounds such as chloramphenicol, chloramphenicolone, morphine, or codeine [117]. On the other hand, it appears that certain compounds can be selectively enriched in PDNVs. Cucumbers-derived nanovesicles possess a high concentration of cucurbitacin B, which effectively inhibited tumor growth, surpassing the efficacy of using cucurbitacin B alone [18]. Similarity, lemons contain various flavonoids, grapefruits contain naringin, ginger contain curcumin and shogaol, apples contain flavonoids, and strawberries contain ascorbic acid [16]. These findings suggest the presence of a potential sorting mechanism between the compounds found in PDNVs and their originating plants.

Interestingly, recent studies have put forward the notion of a mechanism responsible for the enrichment of lipophilic substances within PDNVs, adding to the depth of our understanding [79, 121]. For example, the proportion of PA in whole grapes was found to be significantly lower (18.2 ± 1.9%) compared to nanovesicles isolated from the same batch of grapes (47.2 ± 5.2%) [37]. Similarly, the predominant sterol lipids in arabidopsis leaves-derived extracellular vesicles were sitosterol (62%) and campesterol (22%), whereas in tissues, the proportions were 47% and 13%. This phenomenon of differential distribution of lipids has been observed not only in plants but also in animals and fungi, suggesting its significance as a fundamental sorting mechanism for the contents of extracellular vesicles [17]. However, the precise nature of this sorting mechanism remains elusive, necessitating further investigation, and it is crucial to experimentally verify whether all lipophilic substances are encapsulated and their contents retained.

Gaining a deeper understanding of the intricate relationship between PDNVs and their parent plants is of paramount importance. While metabolites serve as key active components in the mechanism of action of PDNVs, lipids, miRNAs, and proteins in PDNVs also play crucial roles [117, 122]. However, the lack of comprehensive databases has hindered the comparison of lipids, miRNAs, and proteins with their corresponding source plants. By harnessing various high-throughput data with employing bioinformatics approaches, we can fully leverage the available information to establish comprehensive databases and enable the prediction of specific active functions. Identifying patterns and correlations in these datasets can significantly reduce unnecessary exploration in this field and propel research on the underlying sorting mechanisms that govern PDNVs composition.

## Critical consideration on the clinical use of PDNVs

(1) Selection of plant sources and quality control. Currently, research on PDNVs is emerging, and these effects are highly anticipated. However, there are crucial issues regarding the selection of plant sources and quality control that may hinder their clinical applications and need early attention. As mentioned before, nanovesicles derived from different parts of various plants have shown different effects [12]. Moreover, the growth environment of plants, including geographical location, soil conditions, climate, and other factors, significantly influences their composition. Varieties, batches, seasons, and maturity of plants also play a role as influencing factors, affecting batch stability. For example, in terms of liver protection, many PDNVs have demonstrated corresponding effects, but the question remains: which one is more potent? Which ones hold more promise or have stronger advantages to advance into clinical applications?

The isolation methods of PDNVs also impact their composition. The activity of nanovesicles obtained through differential centrifugation and density gradient centrifugation varies. The biological activity of nanovesicles from different centrifugation methods has been confirmed to differ. However, PDNVs, being directly extracted from plant sources as a mixture, appear more complex. Currently, the characterization methods mainly rely on transmission electron microscopy to observe the morphology of vesicles, dynamic light scattering or nanoparticle tracking analysis to detect particle size and distribution. These methods are primarily focused on the physical aspects and cannot serve as quality control standards. Furthermore, due to the complex and diverse sources of PDNVs, there are currently no further characteristic markers available as quality control standards.

(2) Safety considerations. Numerous studies have demonstrated the good biocompatibility of PDNVs through in vitro and in vivo experiments. However, comprehensive preclinical experiments are still lacking, and the following issues need to be considered. The route of administration is one aspect to consider. Currently, PDNVs used in research are of dietary origin, which is reassuring as they are present in our daily food. However, it has been suggested that oral administration may avoid complement system activation, while intravenous administration may activate the complement system [97]. Biological metabolism and long-term safety are another significant concern. Biological metabolism is essential in drug research. Currently, the metabolism of vesicles mainly relies on tracking the fluorescent signals of vesicles using small animal in vivo optical imaging systems. As mentioned earlier, PDNVs are complex compounds containing lipids, proteins, RNA, and secondary metabolites. The specific metabolic processes of these substances in the body have not been extensively studied. Evaluations of biological safety are often short-term, and many studies only focus on studying the active effects without elucidating the underlying mechanisms.

In conclusion, there are still many unresolved questions. Therefore, in this promising stage with numerous emerging effects, more basic research is needed to further investigate PDNVs, including elucidating sorting mechanisms, which will help discover specific markers for quality control. Furthermore, more studies are required to ensure the safety of PDNVs.

## Conclusion

In conclusion, the lipids, proteins, RNAs, and metabolites present within PDNVs play a crucial role in cell recognition, uptake, and exert excellent capabilities in enhancing cell proliferation, migration, and differentiation. The potential of PDNVs to modulate local tissue microenvironments, including promoting angiogenesis, antioxidation, anti-inflammation, anti-bacteria, and anti-aging effects, makes them a compelling avenue for tissue protection and repair. Leveraging these potentials, PDNVs have demonstrated significant protective effects on organs such as the liver, brain, and skin against external stimuli, such as UV radiation, alcohol, and radiation. Moreover, they exhibit substantial reparative effects on conditions like skin defects-induced insulin resistance, colitis, obesity, among others. Additionally, the unique lipid bilayer structure of PDNVs allows for surface modifications and drug loading to further augment their therapeutic effects in tissue protection and repair. It is essential to underscore that appropriate drug delivery strategies are vital for realizing targeted therapeutic effects.

Although we have observed many promising therapeutic impacts, the selection of PDNVs with substantial tissue protection and repair effects remains an area worth exploring. Preliminary evidence from existing research suggests that plant-derived lipophilic compounds may have a role in selecting PDNVs with potent therapeutic effects. Nonetheless, it is paramount to acknowledge that not all efficacious components in PDNVs are compounds, necessitating further database establishment and standardization. Meanwhile, the sources of PDNVs and quality control, as well as the in vivo metabolism and long-term biosafety of PDNVs, are important issues that need to be carefully considered for further application. It is heartening to note that at least two clinical trials on PDNVs are currently in progress (https://clinicaltrials.gov/). Increasing interest in these mini vesicles from researchers suggests a promising future for PDNVs in the realm of tissue protection and repair.

## Data Availability

Data sharing is not applicable to this article as no datasets were generated or analyzed during the current study.
